# Phosphoproteomic analysis of metformin signaling in colorectal cancer cells elucidates mechanism of action and potential therapeutic opportunities

**DOI:** 10.1002/ctm2.1179

**Published:** 2023-02-13

**Authors:** Barbora Salovska, Erli Gao, Sophia Müller‐Dott, Wenxue Li, Carlos Chacon Cordon, Shisheng Wang, Aurelien Dugourd, George Rosenberger, Julio Saez‐Rodriguez, Yansheng Liu

**Affiliations:** ^1^ Yale Cancer Biology Institute Yale University West Haven Connecticut USA; ^2^ Institute for Computational Biomedicine Faculty of Medicine Heidelberg University Hospital Bioquant, Heidelberg University Heidelberg Germany; ^3^ West China‐Washington Mitochondria and Metabolism Research Center West China Hospital Sichuan University Chengdu China; ^4^ Department of Systems Biology Columbia University New York New York USA; ^5^ Department of Pharmacology Yale University School of Medicine New Haven Connecticut USA

**Keywords:** colorectal cancer, DIA mass spectrometry, drug synergy, kinase activity analysis, metformin, network analysis, phosphoproteomics, phosphorylation signature, proteomics

## Abstract

**Background:**

The biguanide drug metformin is a safe and widely prescribed drug for type 2 diabetes. Interestingly, hundreds of clinical trials have been set to evaluate the potential role of metformin in the prevention and treatment of cancer including colorectal cancer (CRC). However, the “metformin signaling” remains controversial.

**Aims and Methods:**

To interrogate cell signaling induced by metformin in CRC and explore the druggability of the metformin‐rewired phosphorylation network, we performed integrative analysis of phosphoproteomics, bioinformatics, and cell proliferation assays on a panel of 12 molecularly heterogeneous CRC cell lines. Using the high‐resolute data‐independent analysis mass spectrometry (DIA‐MS), we monitored a total of 10,142 proteins and 56,080 phosphosites (P‐sites) in CRC cells upon a short‐ and a long‐term metformin treatment.

**Results and Conclusions:**

We found that metformin tended to primarily remodel cell signaling in the long‐term and only minimally regulated the total proteome expression levels. Strikingly, the phosphorylation signaling response to metformin was highly heterogeneous in the CRC panel, based on a network analysis inferring kinase/phosphatase activities and cell signaling reconstruction. A “MetScore” was determined to assign the metformin relevance of each P‐site, revealing new and robust phosphorylation nodes and pathways in metformin signaling. Finally, we leveraged the metformin P‐site signature to identify pharmacodynamic interactions and confirmed a number of candidate metformin‐interacting drugs, including navitoclax, a BCL‐2/BCL‐xL inhibitor. Together, we provide a comprehensive phosphoproteomic resource to explore the metformin‐induced cell signaling for potential cancer therapeutics. This resource can be accessed at https://yslproteomics.shinyapps.io/Metformin/.

## Graphical abstract

- An in‐depth proteomic and phosphoproteomic resource on metformin signaling in colorectal cancer (CRC).
- The phosphoproteomic response to metformin was highly heterogeneous in CRC cell lines.
- Establishing a phosphorylation site‐specific MetScore, which identified 55 most significant metformin signature sites.
- Leveraging the phosphoproteomics to discover potential metformin∼drug interactions in CRC, e.g., navitoclax.

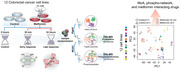

## 1 INTRODUCTION

The biguanide drug metformin is the first‐line treatment for type 2 diabetes (T2D) that has been used in Europe since 1960s and was approved by the United States Food and Drug Administration (US FDA) in the 1990s.^1^ The drug has a superior safety profile and is highly tolerated with a minimum of serious side‐effects compared with other biguanides^2^ and has been used by more than 150 millions of people worldwide.^3^ In addition to the anti‐hyperglycaemic effect, accumulating evidence has suggested multiple beneficial effects of metformin therapy including weight loss in obese patients, cardiorenal protection, neuroprotection and overall health improvement.^4^ Consequently, metformin has been used off‐label to support the treatment of other diverse conditions.^5^ Moreover, a retrospective study published in 2005 reported for the first time that metformin reduced cancer risk in diabetic patients.^6^ Since then, metformin has been investigated in 400 clinical trials (June 2022, ClinicalTrials.gov) for its potential in prevention or treatment of various cancer types.

Colorectal cancer (CRC) has been one of the most prevalent cancer types and one of the leading causes of cancer death globally.^7^ Moreover, a meta‐analysis systematically investigating the association between CRC and T2D revealed a 30% increased risk of developing CRC in diabetic patients.^8^, ^9^ Mounting evidence indicates a potential role of metformin in prevention and improved prognosis.^10^, ^11^, ^12^, ^13^, ^14^, ^15^, ^16^ For example, a recent meta‐analysis reported metformin use decreased the risk of CRC in T2D patients by 29% and reduced both all‐cause mortality of CRC in T2D and CRC‐specific mortality in T2D.^17^ Furthermore, in in vitro models of CRC, metformin has shown synergistic effects with oxaliplatin,^18^ cisplatin,^19^ irinotecan^20^ and fluorouracil,^21^ highlighting metformin as a potential sensitisation agent to CRC chemotherapy. Similar observations have been made for other cancer types.^22^, ^23^, ^24^ On the other hand, the effects of metformin on overall and recurrence‐free survival in CRC patients undergoing chemotherapy after resection remain inconclusive including both studies suggesting a positive effect^25^, ^26^, ^27^, ^28^ and no effect of metformin.^29^, ^30^

One possible mechanism of action (MoA) of metformin is the inhibition of mitochondrial respiratory complex I leading to activation of the 5′‐AMP‐activated protein kinase (AMPK) in a serine/threonine‐protein kinase STK11 (LKB1)‐dependent manner.^31^ The activation of AMPK is followed by serine/threonine‐protein kinase mTOR (mTOR) inhibition leading to favourable phenotypic outcomes in cancer cells, such as reducing protein synthesis and proliferation rates, activation of autophagy and inhibition of inflammatory responses.^23^ Thus, AMPK appears to be a widely accepted target of metformin. However, AMPK can be also activated by other kinases, such as by the calcium‐sensitive kinase calcium/calmodulin‐dependent protein kinase kinase 2/beta (CAMKK2)^32^, ^33^, ^34^ or mitogen‐activated protein kinase kinase kinase 7 (MAP3K7; TAK1).^35^ Furthermore, metformin can affect cellular signaling in an AMPK‐independent manner.^36^ Therefore, the MoA underlying metformin's gastrointestinal anti‐cancer properties seems pleiotropic and remains largely mysterious.^4^, ^23^, ^24^, ^37^

The mass spectrometry (MS)‐based techniques may facilitate the discovery of MoA of drugs via, for example, phosphoproteomic profiling.^38^ Regarding the MoA of metformin, a previous phosphoproteomic study in one breast cancer cell line (MCF7) showed that the anti‐cancer activity of metformin is not mediated by a limited number of isolated signaling cascades but rather complex.^39^ In another study profiling protein levels, AMPK‐dependent and independent kinome perturbation in mouse liver cells treated by metformin uncovered novel kinases mediating hepatic metabolism.^36^ As an arising MS technique, data‐independent acquisition mass spectrometry (DIA‐MS)^38^, ^40^, ^41^ generates continuous, high‐resolution MS2 peak profiles along with liquid chromatography (LC) separation and enables simultaneous identification, localisation and quantification of phosphorylation sites (P‐sites).^42^, ^43^, ^44^, ^45^ Herein, we applied a DIA‐MS‐based workflow to perform deep and highly quantitative proteome and phosphoproteome profiling of a panel of 12 highly heterogeneous CRC cell lines treated with metformin. Our study presents a considerable phosphoproteome resource revealing and rationalising heterogeneity of metformin responses in CRC cells, reinforcing the knowledge of metformin MoA towards cancers therapeutics. We provide this resource in a form of an interactive website (https://yslproteomics.shinyapps.io/Metformin/) enabling further exploration of metformin MoA by the scientific community.

## 2 MATERIAL AND METHODS

### 2.1 Cell culture and metformin treatment

The following 12 CRC cell lines were used in this study: C2BBe1, COLO 205, HT115, LoVo, MDST8, NCI‐H747, RKO, SNU‐61, SW48, SW837, SW848 and T84. The cells were cultured on 10 cm dishes at 37°C, humidified 5% CO_2_ in a complete medium containing L‐glutamine, supplemented with 10% foetal bovine serum (Gibco; #26140079) and penicillin–streptomycin (Sigma–Aldrich; #P0781). The following cell culture media were used: Dulbecco's modified Eagle medium (DMEM; Corning; #10‐013‐CV) was used for C2BBe1, HT115 and MDST8; RPMI‐1640 (Thermo Scientific; #11875‐093) was used for COLO 205, LoVo, NCI‐H747, RKO, SNU‐61 and SW48; Leibovitz's L‐15 Medium (Cytiva; #SH0525.01) was used for SW837 and SW948; DMEM/F12 (Thermo Scientific; #11330‐032) was used for T84.

2 × 10e^6^ – 4 × 10e^6^ cells were seeded in a 10 cm dish, cultured for 24 h and then treated with 10 mM metformin as described in a previous metformin phosphoproteomic study.^39^ The following samples were harvested in three replicates: control samples at time points 0 and 24 h and metformin‐treated samples at time points 30 min and 24 h. The 30‐min time point was skipped for the total proteome analysis. After washing twice in PBS, the dishes were snap‐frozen in liquid nitrogen for 2 min, and the cells were scraped into 200 μl of cell lysis buffer containing 10 M urea/ 100 mM ammonium bicarbonate, cOmplete™ protease inhibitor cocktail (Roche; #11697498001) and the Halt phosphatase inhibitors (Thermo Scientific; #78428). Cells were scraped using a cell scraper into a 2 ml Eppendorf tube. Tubes were vortexed for 30 s, snap‐frozen in liquid nitrogen and transferred to store at −80°C until further sample processing. Triplicate dishes per cell line and condition were used as three whole process replicates for the analysis. After collecting all samples, the samples were randomised prior further processing as shown in Table S2 and S3.

### 2.2 Protein extraction and digestion

Cell pellets in lysis buffer were thawed and sonicated twice at 4°C for 1 min using the VialTweeter device (Hielscher‐Ultrasound Technology)^46^ and centrifuged at 20,000 g for 1 h to remove insoluble material. Protein concentration in the supernatant was determined using the Bio‐Rad protein assay dye (Bio‐Rad; #5000006). The protein samples (800 μg of protein per sample) were diluted in 6 M urea/100 mM ammonium bicarbonate buffer to 400 μl (final concentration = 2 μg/μl), reduced by 10 mM DTT for 1 h at 56°C and alkylated by 20 mM IAA in dark for 1 h at room temperature (RT). The reduced and alkylated proteins were then subjected to a precipitation‐based digestion as described previously.^47^ Briefly, five volumes of precooled precipitation solution containing 50% acetone, 50% ethanol and 0.1% acetic acid were added to the protein mixture and kept at −20°C overnight. The mixture was centrifuged at 20,000 × *g* for 40 min. The precipitated proteins were washed with precooled 100% acetone and centrifuged at 20,000 × *g*, 4°C for 40 min. After acetone aspiration, the remaining acetone was evaporated in a SpeedVac. Next, 300 μl of 100 mM NH_4_HCO_3_ with sequencing grade porcine trypsin (Promega) at a ratio of 1:20 were added and incubated overnight at 37°C. The resulting peptide mixture was acidified with formic acid and then desalted with a C18 column (MarocoSpin Columns, NEST Group INC.) following the manufacturer's instructions. The amount of the final peptides was determined by a nanodrop (Thermo Scientific).

### 2.3 Phosphopeptide enrichment

The phosphopeptide enrichment was performed using High‐Select™ Fe‐NTA kit (Thermo Scientific; #A32992) according to the kit instructions, as described previously.^48^ Briefly, the peptide‐resin mixture was incubated for 30 min at RT while gently shaking and then transferred into a filter tip (TF‐20‐L‐R‐S; Axygen) to remove the supernatant (flow‐through) by centrifugation. The resins were washed three times with 200 μl of washing buffer (80% I, 0.1% TFA) and twice with 200 μl of H_2_O. The phosphopeptides were eluted twice with 100 μl of elution buffer (50% ACN, 5% NH_3_•H_2_O) and dried in SpeedVac (Thermo Scientific). The amount of the final phosphopeptides was determined by a nanodrop (Thermo Scientific).

### 2.4 Mass spectrometry measurements

For the LC–MS analysis, 1 μg of peptide and phosphopeptide mixture was used as described previously.^46^, ^49^ The LC separation was performed using an EASY‐nLC 1200 systems (Thermo Scientific) and a PicoFrit column (New Objective, Woburn, MA, USA; 75 μm × 50 cm length) self‐packed with ReproSil‐Pur 120A C18‐Q 1.9 μm resin (Dr. Maisch GmbH, Ammerbuch, Germany). A 150‐min measurement with buffer B (80% acetonitrile containing 0.1% formic acid) from 5 to 37% and corresponding buffer A (0.1% formic acid in H_2_O) during the gradient was used to elute peptides from the LC. The flow rate was set to 300 nl/min with the temperature controlled at 60°C using a column oven (PRSO‐V1; Sonation GmbH, Biberach, Germany). The Orbitrap Fusion Lumos Tribrid mass spectrometer (Thermo Scientific) was coupled with a NanoFlex ion source keeping the spray voltage at 2000 V and heating capillary at 275°C. The DIA‐MS method consisted of a MS1 survey scan and 33 MS2 scans of variable windows as described previously.^50^, ^51^ The MS1 scan range is 350–1650 m/z and the MS1 resolution was 120,000 at m/z 200. The MS1 full scan AGC target value was set to be 2.0E6, and the maximum injection time was 100 ms. The MS2 resolution was set to 30,000 at m/z 200, and the normalised HCD collision energy was 28%. The MS2 AGC was set to be 1.5E6, and the maximum injection time was 50 ms. The default peptide charge state was set to 2. Both of MS1 and MS2 spectra were recorded in a profile mode.

### 2.5 Proteomic and phosphoproteomic DIA data analysis

DIA‐MS data analyses for proteomics and phosphoproteomics were performed using Spectronaut v14^50^, ^52^ using the library‐free directDIA pipeline^50^, ^53^ as described in detail in our previous study.^43^ The DIA runs were all directly searched against the Swiss‐Prot protein database (September 2020, 20,375 entries). For the total proteome dataset, methionine oxidation and N‐terminal acetylation were set as variable modifications, and carbamidomethylation at cysteine was set as a fixed modification. Additionally, for the searching of the phosphoproteomic dataset, phosphorylation at serine/threonine/tyrosine (S/T/Y) was enabled as a variable modification. Both peptide‐ and protein‐FDR were controlled at 1%, and the resulting data matrix was filtered by ‘Qvalue’. The PTM localisation function in Spectronaut v14 was enabled to localise and filter the phosphorylation sites using a PTM score > .75.^42^, ^44^ All the other Spectronaut settings were kept as default, for example, the ‘Interference Correction’ was enabled, the ‘Global Normalization’ (‘Median’) was used, the quantification was performed at the MS2 level using peak areas and the Top 3 peptide precursors (‘Min: 1 and Max: 3’) were averaged (mean) for representing protein quantities in all DIA analyses.

### 2.6 Data processing

For the total proteome analysis, the protein pivot report was exported from Spectronaut. Relative intensity values < 500 were replaced by NA, and the data were log2 transformed and normalised using LOESS.^54^ For the phosphoproteome data analysis, phosphopeptide precursor level pivot report was exported from Spectronaut using two different localisation probability score cutoffs (0.75 and 0) using a strategy described in our previous work.^43^ The first report (localisation probability > .75, class I sites,^55^ *n* = 117,067 unique modified peptide precursor ions) was used to identify phosphopeptide precursor ids with confidently localised P‐sites in at least one sample. The second report (localisation probability > 0, *n* = 132,887 unique modified peptide precursor ions) was filtered to only contain the 117,067 sites from the first report and was used to extract the phosphopeptide precursor intensity values. The resulting total number of identified unique phosphoprecursors was 92,422. To select the most representative phosphopeptide precursor per unique phosphopeptide id, ‘phos.id’, we applied the following filtering criteria: (i) the precursor with the most valid values across the 144 MS runs, (ii) in case there were multiple precursor ids with the same number of valid values, the top intensity id was used (sum intensity across samples). The number of resulting unique phos.ids was 64,680 from 7250 phosphoproteins; these ids preserved information about multiply phosphorylated peptides and were used for the downstream analysis. These ids corresponded to 56,080 unique P‐sites (i.e., protein + site localisation, ‘phos.id.exp’) and were used to perform the data annotation for all site‐specific analyses. Phosphopeptide intensities were also filtered to remove all intensities < 500, log2 transformed and normalised using LOESS.^54^

### 2.7 Detection of net phosphorylation quantitative changes

When specified, the relative protein abundances were regressed out from the respective relative phosphopeptide abundance values to detect net phosphorylation changes using linear regression as described previously.^56^ Briefly, phosphopeptide intensities were set as the dependent variables (*y*), and matched protein intensities were set as the independent variables (*x*). The residuals of the *y*∼*x* linear model were used as the phosphorylation levels not driven by protein abundance levels.

### 2.8 Consensus clustering

Consensus clustering^57^, ^58^ was performed with the ConsensusClusterPlus R package^58^ using log2 fold changes (metformin 24 h/control 24 h) or normalised log2 intensities (‘steady‐state’). Only those ids with quantitative values in all cell lines were used, and the top 30% most variable ids were selected based on their variability across samples (median absolute deviation; MAD). The number of clusters selected for metformin response classification was four.

### 2.9 Statistical analysis

Statistical analysis to identify differentially abundant proteins and phosphopeptides was performed in Perseus v1.6.14.0^59^ using a two‐sided Student's *t*‐test for every comparison with at least 2 valid values per each compared group to avoid missing values imputation. Protein groups and phos.ids with *p* < .01 and a fold‐change > 1.5 were reported as significant. To identify differentially abundant phos.ids at 30 min and 24 h, the control samples at 0 and 24 h were used, respectively, to consider the effect of nutrient exhaustion in media on the phosphoproteome after 24 h of cultivation. To identify differentially abundant proteins after 24 h, we used the matched 24h control samples as well.

### 2.10 MetScore calculation

To identify phos.ids consistently responding to metformin treatment across the 12 cell lines, we developed a scoring system which we termed MetScore. After statistical analysis (*p* < .01 and fold‐change > 1.5; two‐sided Student's *t*‐test) only those ids subjected to the statistical analysis in all cell lines (i.e., having required number of valid values as described above) were used, leading to a complete matrix containing 14,032 unique phos.ids for the 24‐h dataset. The score was calculated across the 12 cell lines, that is, when a phos.id was significantly up‐regulated, we added one (+1) to the score, and when an id was significantly down‐regulated, we subtracted one (−1) from the resulting score, leading to a score distribution from −12 to 12 reflecting the consistency of the response across the cell lines. Based on the MetScore, the phos.ids were stratified into five segments for the downstream enrichment analysis: MetScore G1 (MetScore ϵ [4, 12], *n* = 1207, 8.6% of the dataset), MetScore G2 (MetScore ϵ [3, 2], *n* = 1660, 11.83%), MetScore G3 (MetScore ϵ [−1, 1], *n* = 8344, 59.86%), MetScore G4 (MetScore ϵ [−2, −3], *n* = 1,607, 11.45%) and MetScore G5 (MetScore ϵ [−4, −12], *n* = 1214, 8.65%).

### 2.11 Sequence analyses

To compare the MetScore G1 and G5 P‐sites sequence windows (Figure 4D), we used iceLogo^60^ with *p* < .05 cutoff. To extract enriched sequence motifs (Figure 4E), we employed motifeR^61^ with a minimal occurrence of a motif set to 20 and the motif enrichment *p* < .000001.

### 2.12 Data annotation and functional enrichment analyses

Protein‐specific annotation was downloaded from DAVID Bioinformatics Resources 6.8 [https://david.ncifcrf.gov/].^62^, ^63^, ^64^ Site‐specific annotation was downloaded from the PhosphoSitePlus database [www.phosphosite.org],^65^, ^66^ PTMsigDB v1.9.0^67^ and the OmniPath database.^68^, ^69^ The validated lysosomal metformin‐interacting protein list used in Figure 3 (*n* = 113) was downloaded from the recent Ma et al. publication.^70^ The functional score used in Figure 4 was downloaded from the Ochoa et al. publication.^71^ The 1D enrichment and Fisher's exact test analyses were performed in Perseus v1.6.14.0^59^ or in R.^72^ For the protein‐specific annotation enrichment analyses of the phosphoproteome dataset, the analyses were performed relative and not relative to unique protein ids to include the information about the number of phosphorylation events per protein in a pathway but also consider the number of unique protein ids identified per pathway. For the 1D enrichment analyses using fold changes between conditions (such as in Figure 2E), a looser filtering was applied to the data as follows. First, the replicate values were averaged (mean) for each sample requiring at least one value per triplicate, and then a fold‐change was calculated between conditions leading to a more complete matrix containing more P‐sites for a more sensitive biological analysis. The purpose of this ‘looser’ filtering (i.e., also the usage of ratios based on the quantification in at least one replicate per condition) is to improve the data completeness by relying on the *de facto* ratios measured between conditions and to facilitate functional enrichment analysis, which normally needs more proteins in the input lists.

### 2.13 Kinase activity estimation and signaling network modelling

After data processing and normalisation, differential analysis was performed between each control and metformin‐treated cell line after 24 h using the limma R‐package.^73^ The standard sequence of lmFit, contrasts.fit, and eBayes was used, and the limma results were corrected for multiple testing using FDR correction (Benjamini‐Hochberg method). For each cell line, kinase activity was estimated based on the abundance of the direct target P‐sites. Post‐translational modifications were extracted from OmniPath^68^, ^69^ and filtered to keep only phosphorylation and dephosphorylation events. Interactions reported exclusively in the ProtMapper database were removed after noticing inconsistent interactions leading to a total of 29,445 signed kinase‐P‐site interactions of 580 different kinases. Additionally, *t*‐values from the differential analysis of each P‐site after metformin stimulation using the limma R‐package were passed to the run_viper function from the decoupleR R package.^74^, ^75^ Only kinases with at least five measured targets were included in the kinase activity estimation.

Signaling networks were contextualised for each cell line using the R‐package PHONEMeS^76^ (PHOsphorylation NEtworks for Mass Spectrometry) by combining the information from large‐scale MS phosphoproteomic data with prior knowledge of signaling. Based on integer linear programming implementation for causal reasoning, PHONEMeS finds a path that connects deregulated P‐sites with deregulated kinases in a coherent manner. For that a selection of deregulated P‐sites and deregulated kinases and a prior knowledge network (PKN) are required. The PKN contains protein–protein and kinase‐P‐site interactions retrieved from OmniPath, resulting in a network with a total of 49,219 edges and 18,966 unique nodes. The top 15% of P‐sites based on their absolute *t*‐value were selected as deregulated and their *t*‐values were passed to PHONEMeS. For the selection of deregulated kinases, the top 15% based on their absolute normalised enrichment score (NES) were chosen and selected as up‐regulated (1) with a NES over zero and down‐regulated (−1) with a NES lower than zero. For cell lines where AMPK (PRKAA1) belonged to the top 15% based on its absolute NES, this node was manually removed from the selected kinases, to be able to gain insights about its upstream regulation. Additionally, kinases with an absolute NES lower than 0.5 were passed to PHONEMeS to be removed from the PKN. Before connecting deregulated P‐sites to kinases, the PKN was pruned by removing all nodes that are not connected 50 steps downstream and upstream of the selected kinases and P‐sites, respectively. For each node in the resulting networks the inferred activity of PHONEMeS was compared with the estimated activity from decoupleR if possible. If these activities did not agree on their direction of deregulation, the nodes were either removed from the PKN (if NES < 2) or added to the inputted deregulated kinases (if NES ≥ 2). PHONEMeS was re‐run with the adapted inputs and all steps were repeated as described above until a network solution was found where the PHONEMES‐inferred and decoupleR‐estimated activities were coherent for all overlapping nodes. Protein–protein networks were constructed for each cell line by removing interactions between kinases and phosphorylation sites from the network. To focus on regulation surrounding PRKAA1, subnetworks were extracted containing all nodes and edges two steps up‐ and downstream of PRKAA1. For the comparison of subnetworks between different cell lines, a general backbone was created containing the nodes of all subnetworks, with cell line‐specific nodes highlighted.

### 2.14 Drug signature comparison

To identify drugs potentially interacting with metformin CRC, we queried the ATLANTiC database^77^ [http://atlantic.proteomics.wzw.tum.de] for the 55 metformin signature sites (absolute MetScore ≥ 10). For each site, we extracted the top 50 most significant drug associations based on ‘Simple correlation’ analysis *p* value. Using these drug∼P‐sites associations, we generated a network and finally selected drugs shown in Figure 7 based on their degree (number of edges). The correlation analysis presented in the Supplementary material was performed as follows, similarly to a previous study.^78^ Drug signature comparisons were performed by querying Touchstone‐P, a library of phosphoproteomic signatures of the relative abundances of approximately 100 phosphorylation sites (P100)^79^ from a panel of 7 different cell lines treated with 118 small‐molecule drugs^80^, ^81^ available through the ConnectivityMap web interface (http://clue.io/proteomics‐query). The input P100 phosphorylation signatures of the 12 CRC cell lines treated with metformin for 24 h were compared with each signature in the library a connectivity score ranging from −1 (strong negative connection/most ‘opposite’ profile) to 1 (strong positive connection/most similar profile) was reported for further analysis. The second analysis was performed using PhosFate Profiler^82^ [http://phosfate.com]. Briefly, the relative log2 fold changes induced by metformin in individual cell lines were submitted to retrieve the correlation of the signaling response with 399 previously published phosphoproteomes.

### 2.15 Data visualisation

Most data visualisation was performed in R.^72^ The following R packages were used to visualise the data: ‘ggplot2’ (boxplots, violin plots, volcano plots and histograms), ‘plot’ (principal component analysis [PCA]), ‘pheatmap’ (heatmaps), ‘corrplot’ (correlation plots) and ‘UpsetR’ (UpSet plots). Barplots were generated using GraphPad Prism version 8.0 (GraphPad Software, San Diego, California USA). The kinome trees were generated using Coral.^83^ All networks were visualised in Cytoscape v3.9.1.^84^

### 2.16 Drug combination cell viability assays

LoVo, SNU‐61 and SW948 cell lines were seeded into 96‐well plates (3000–5000 or 10,000 per well for the 24 and 72‐h assay respectively) for 24 h before adding 10 mM metformin without refreshing the media. After 24 h of pre‐incubation with metformin, the second drug was added in a dilution series of six different concentrations, and the viability of the cells was assayed after 24 and 72 h of the combined drug treatment using a fluorescence‐based resazurin proliferation assay.^85^, ^86^ Resazurin (30 μg/ml) was added to growth media, and fluorescence signal (excitation wavelength 560 nm, emission wavelength 590 nm) was measured after 3 h using a BioTek synergy 2 plate reader (Agilent, Santa Clara, CA, USA). Loewe synergy score and the corresponding *p* values were calculated using the SynergyFinder R package.^87^

### 2.17 Mass spectrometry data availability

The mass spectrometry data have been all deposited to the ProteomeXchange Consortium via the PRIDE with the dataset identifier PXD036826.^88^

## 3 RESULTS

### 3.1 A high‐quality mass spectrometry dataset captured the acute and late metformin response in CRC

**FIGURE 1:**
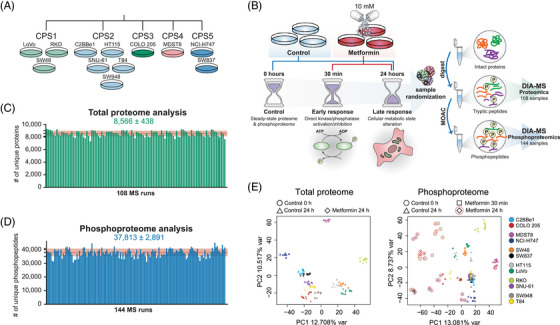
Proteomic and phosphoproteomic analysis of metformin‐treated colorectal cancer cells. (A) Twelve CRC cell lines selected for the study represent 5 proteomic clusters (CPS1‐CPS5).^56^ (B) The cell lines in triplicate dishes per condition were seeded 24 h prior metformin treatment and harvested after 30 min and 24 h to capture the acute and late responses to metformin. After harvesting all samples, the 144 conditions in triplicates were randomised prior sample processing, sample preparation and LC–MS analysis. Matched tryptic peptides and enriched phosphopeptides were subjected to DIA‐MS. Data were analysed using the library‐free algorithm directDIA in Spectronaut v14. The PTM workflow in Spectronaut was applied. (C and D) The numbers of identified unique protein groups (C) and unique phosphopeptides (protein and peptide FDR < 1%) with confidently localised P‐sites (i.e., class I sites; D) across a total of 252 (i.e., 108+144) randomised injections. (E) Principal component analysis of all total proteome (left) and phosphoproteome (right) samples (*n* = 3 replicates per condition).

To interrogate the total proteome and phosphoproteome response to metformin in CRC cells, we firstly aimed to select cell lines representing heterogeneous CRC subtypes (Figure 1A). Previously, Roumeliotis et al. analysed 50 CRC cell lines based on their proteomic profiles and identified five proteomic subtypes (CPS1‐CPS5), which overlapped well with the clinical CRC tissue subtypes.^56^, ^89^, ^90^, ^91^ Among those subtypes, CPS1‐3 represent groups classified based on microsatellite instability, hypermutation state, ABC transporter expression and other factors. The CPS4‐5 subtypes represent the colorectal stem‐like subgroup.^56^ In the present study, we therefore selected 12 cell lines to represent these five clusters: three from CPS1 (LoVo, RKO, SW48), five from CPS2 (C2BBe1, HT115, SNU‐61, SW948, T84), one from CPS3 (COLO 205), one from CPS4 (MSDT8) and two from CPS5 (NCI‐H747, SW837). The cancerous mutational statuses of these cell lines are summarised in Table S1.^89^ Next, following a published investigation by Sacco et al. in the MCF7 cell line,^39^ we cultured the CRC cells for 24 h before the treatment with 10 mM metformin (Figure 1B) without refreshing the media, to achieve a partial ‘nutrient exhaustion’ state that could be essential to observe AMPK activation.^39^ Unlike Sacco et al., who focused on a 24‐h treatment, we included two time points post metformin addition: 30 min for unveiling any potential ‘acute’ phospho‐signaling,^70^, ^92^, ^93^ as well as 24 h for investigating the ‘late’ phosphorylation response to metformin.^39^ We did not measure the proteomic changes at 30 min, because such a time window is usually too short for observing any significant proteomic abundance changes in cancer cells. Thus, together with the time point 0 (i.e., the ‘steady‐state’) controls, a total of 108 and 144 samples were collected including three biological replicates per condition. The samples were assigned random order as shown in Table S2 and S3, processed and measured by a single‐shot DIA‐MS method.^43^, ^45^

Based on a library‐free directDIA algorithm^50^, ^53^ (see *Methods*), we identified 10,142 protein groups (8566 ± 438 on average; Figure 1C) and 64,680 unique phosphopeptides (37,813 ± 2891 on average; Figure 1D) corresponding to 56,080 unique class I phosphorylation sites (or, P‐sites, with localisation probability > .75^42^, ^55^) from 7450 unique protein groups in the entire experiment, with the peptide‐ and protein‐FDR strictly controlled below 1% (Tables S2 and S3). Compared with the landmark paper of Sacco et al. in the year of 2016 (performed in a different experimental system including MS acquisition method and platform), our phosphoproteomic‐DIA (Phos‐DIA) profiled 354.6% of their number of P‐sites, significantly extending the analytical depth by using the current MS and bioinformatic platforms.^39^ The median absolute Pearson correlation was .93 and .88 within replicates for the proteome and phosphoproteome analysis, respectively. Both hierarchical clustering analysis (HCA; Figures S1 and S2) and PCA (Figure 1E) confirmed the excellent clustering among biological replicates. To summarise, we acquired deep and quantitative DIA‐MS datasets capturing the acute and late metformin signaling in 12 representative CRC cells.

### 3.2 The impact of metformin is much more evident in the phosphoproteome of ‘late’ response

**FIGURE 2:**
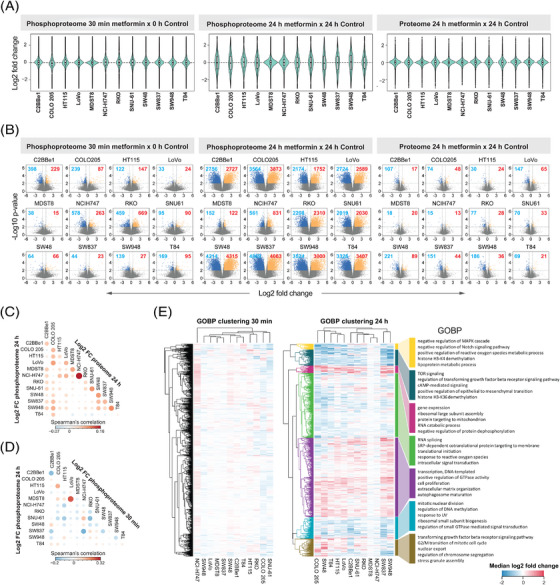
Metformin response occurred predominantly at the phosphoproteome level after 24 h. (A) Violin plots depict the log2 fold change (metformin vs. corresponding control) distribution. (B) Volcano plots show the log2 fold change distribution and statistical significance of phosphopeptides and proteins. The blue and red numbers indicate the number of significantly regulated P‐sites or proteins per cell line and time point (*p* < .01, absolute fold change > 1.5; two‐sided *t*‐test). (C) Correlation plot between the proteome and phosphoproteome level log2 fold changes after 24 h. (D) Correlation plot between the 30‐min and 24‐h log2 fold changes (log2 FC) at the phosphoproteome level. (E) Hierarchical clustering analysis of gene ontology biological process terms (David GOBP Direct). The processes were prefiltered to at least include five unique phosphoprotein ids in each cell line and significantly up‐ or down‐regulated in at least one cell line (1D enrichment analysis in Perseus 1.6.14.0), and then a list of the five most representative terms were selected based on biological relevance and removal of redundant terms. The colour illustrates median log2 fold change per GOBP in each cell line based on all phosphoproteins annotated by a GOBP. The same set of GOBPs in the 30 min (left) and 24 h (right) heatmaps are shown. The right panel shows selected significant GOBP terms per GOBP cluster based on the 24 h data

How prevalently metformin affects protein expression? Based on our measurement, the proteome level change was minor after 24 h in all CRC cell lines, especially when compared with the phosphoproteome that was extensively rewired at the same time point (Figures 2A and B). Collectively, across all CRC cell lines, while 20.9 ± 12.1% (mean ± SD) of the phosphoproteome was significantly (*p* < .01 & absolute fold change > 1.5) regulated after 24 h (Figures 2A and B, middle panel), only 1.7 ± 1.1% of the proteome underwent significant regulation (Figures 2A and B, right panel). In accordance, in PCA, the largest variability in the cell line panel at the proteome level was due to the heterogeneity of the baseline proteomes rather than the treatment. In contrast, at the phosphoproteome level, the most variability was explained by different treatment conditions (Figure 1E). It should be noted that, the ‘control’ cells in non‐refreshed media for 24 h tended to exhibit a global separation from the time point 0 phosphoproteome samples with AMPK activation (demonstrated by increased phosphorylation of several AMPK targets; Figure S3). This result confirmed the existence of the ‘partial nutrient exhaustion state’ as described previously^39^ as well as the importance of using a time point‐matched control. Correlation analysis (Figure 2C) indicated a very weak positive correlation (*R* = .02–.16) between the proteome and phosphoproteome levels, suggesting that in this case the phosphoproteome change was not driven by the total proteome change (which is anyway barely detectable) even for the long‐term response. Moreover, subtracting the total protein abundance variation from the phosphopeptide variation using linear regression^56^ did not show any major impact on the metformin‐response quantification (Figure S4). Therefore, we decided to directly use the phosphoproteomic data, whenever applicable, for the downstream signaling analyses. Altogether, our data suggest that metformin only minimally regulates the total proteome expression levels in CRC cell lines.

Previous studies have suggested the existence of an acute response to metformin treatment.^70^, ^92^, ^93^ However, phosphoproteomic characterisation of the rapid response has been lacking. As shown in Figures 2A and B (left panel), we identified differential P‐sites following the 30‐min treatment underscoring an ‘acute’ response of a fraction of the phosphoproteome. However, both the magnitude of the change and the number of significant phosphopeptides were markedly lower compared with the ‘late’ response, after 24 h. Specifically, we found the most prominent acute phosphorylation response in RKO, NCI‐H747 and C2BBe1. Interestingly, for any of these cell lines, the acute and late response did not correlate (Figure 2D) and affected a distinct set of P‐sites (Figure S5). The low extent of phosphoproteome perturbation after 30 min was also reflected at the level of signaling pathways modulated by metformin treatment (Figure 2E, left panel). These results are suggestive of distinctive temporal patterns in the rapid metformin response of particular CRC cells. AMPK activation and mTOR inhibition have been referred as ‘hallmarks of metformin treatment’ in cancer.^39^ Importantly, we observed an expected up‐regulation and down‐regulation of the phosphorylation of selected known AMPK and mTOR substrates,^94^ respectively, in the late 24‐h response (Figure S6 and S7). These included known mTOR P‐sites Ser 1261, Ser 2478 and Ser 2481 with regulatory functions (Figure S8). Some of these P‐sites, for example, RPTOR Ser 873, showed a minor, but significant change after 30 min, but in general, most of these P‐sites were not affected at 30 min.

In summary, the dominant metformin‐induced response occurred at the phosphoproteome level rather than at the proteome expression level in CRC, and a long‐term 24‐h treatment showed a more pronounced phosphoproteomic perturbation.

### 3.3 The P‐site response to metformin is highly heterogenous among CRC cells

The 12 cell lines were selected to represent heterogenous CRC genotypes and signaling repertoires.^56^ We thus interrogated how the cell line variability affects the metformin response after 24 h of the treatment. *First*, the fold change distributions (Figure 2A) and volcano plots (Figure 2B) distinguished between two groups of cells – in most (10 of 12) cell lines, we identified thousands of differentially (*p* < .01 and absolute fold change > 1.5) phosphorylated peptides (∼13.8–41.9% of the phosphoproteome measured per cell line), while in the other two cell lines, MDST8 and NCI‐H747, we discovered only small extent of phosphorylation change at 24 h (0.8 and 4.1%). Furthermore, even for the former 10 cell lines, the overlaps of differential P‐sites were rather minor (0.08% of the measured phosphoproteome across cell lines, Figure S9). *Second*, we performed a consensus clustering analysis using the top 30% most variable phosphopeptides based on metformin induced log2 fold change for all cell lines. We identified 4 consensus clusters (Figures S10A–C), which did not resemble the clusters identified in the steady‐state proteome and phosphoproteome data (Figure S10D), suggesting that the heterogenous metformin response is not reflected by the basal state of the cells. *Third*, we sought to investigate the heterogeneity of the biological processes induced by metformin between cell lines. Based on the functional enrichment analysis (Figure 2E), processes such as gene expression, RNA splicing, and translational initiation were enriched in up‐regulated direction in most of the cell lines (in all cell lines 1D enrichment *p* values < .05, see *Methods*), indicating that CRC cells prefer to trigger these pathways to cope with the metformin‐altered cellular state. On the other hand, among the processes with down‐regulated phosphorylation, TOR signaling, mitotic nuclear division or regulation of DNA methylation were processes significantly enriched in most of the cell lines. However, at least half of all the processes did not follow the same trend among the 12 cell lines (Figure 2E).

In conclusion, our data suggests that while most cell lines altered their phosphoproteomes as a result of metformin perturbation, the degree of the alteration, the specific phosphorylation sites and the biological processes triggered or inhibited were highly variable across the CRC panel.

### 3.4 The lysosomal impact of metformin according to quantitative proteomics and phosphoproteomics

**FIGURE 3:**
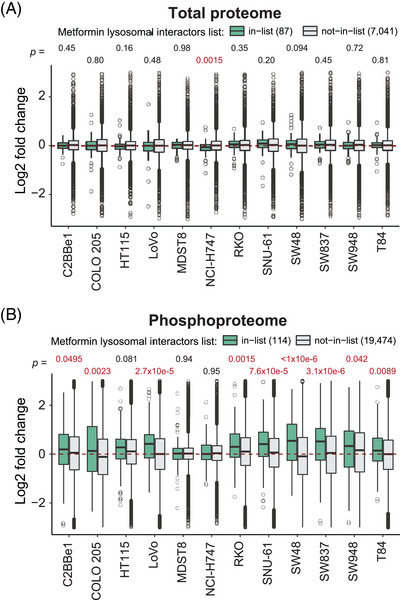
Metformin‐interacting lysosomal proteins tend to be increasingly phosphorylated after 24 h. (A) The total proteome and the (B) phosphoproteome log2 fold changes induced by metformin treatment. The list of metformin‐interacting lysosomal proteins (green boxes) is based on a recently published dataset.^70^ Statistical analysis was performed using the Wilcox test. The list of proteins and P‐sites overlapping with the ‘Ma list’ are provided in Table S4.

A recent study identified key lysosomal metformin–protein interactors, elucidating the mechanism of the lysosomal glucose‐sensing pathway leading to AMPK activation.^70^ Although we did not measure the metformin–protein interactions directly in the present study, we mapped our quantitative proteome and phosphoproteome data to the metformin interacting lysosomal protein list reported in Ma et al. (*n* = 87, hereafter, ‘Ma list’; see *Methods* and Table S4). Intriguingly, while the protein abundances of Ma list identities did not change post metformin treatment (Figure 3A), in nine out of 12 cells we found the up‐regulation of the P‐sites (*n* = 114) for the Ma list proteins (*p* = .0495 to 3.1e−06) upon the 24‐h drug treatment (Figure 3B). This result thus implicates that metformin‐interacting proteins might have increased phosphorylation levels, propagating to subsequent signaling transduction.

### 3.5 Establishing MetScore, a score summarising metformin association for each P‐site

**FIGURE 4:**
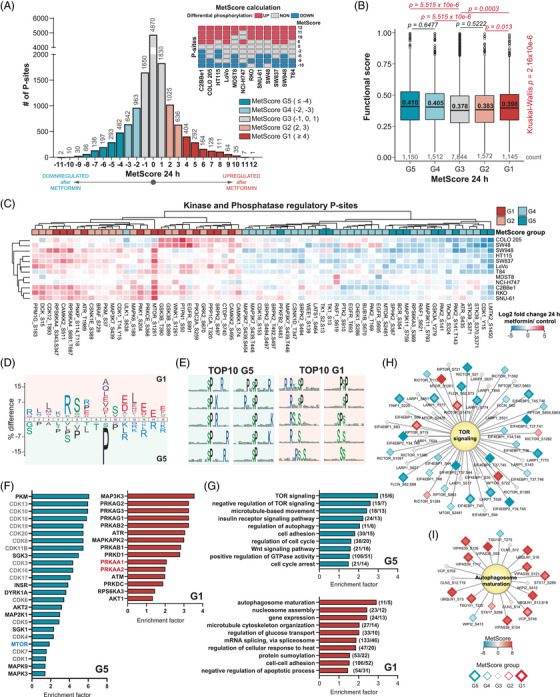
Identification and functional characterisation of the common metformin phosphorylation signature using MetScore. (A) MetScore was calculated for each P‐site based on the results of the statistical analysis (two‐sample *t*‐test *p* < .01 & absolute fold change > 1.5) as a sum of cell lines in which a P‐site was up‐regulated (+1), non‐regulated (+0) or down‐regulated (−1). P‐sites with missing statistical analysis results (i.e., with less than 2 valid values per group) were excluded. Histogram shows the MetScore distribution of all P‐sites (*n* = 14,032). The P‐sites were divided into five segments for a subsequent functional analysis. (B) Distribution of the *P‐site specific functional scores* based on a previous publication by Ochoa et al.^71^ in the MetScore segments. Statistical analysis was performed using Kruskal‐Wallis test; the pairwise comparisons were performed using pairwise Wilcox test with Benjamini–Hochberg correction. (C) Hierarchical clustering analysis of the ‘regulatory’ P‐sites (based on PhosphoSitePlus database) from kinases and phosphatases with an absolute MetScore ≥ 2. (D) Sequence analysis was performed using iceLogo comparing the relative frequencies of amino acids at different positions in the sequence window between the G1 group (foreground/top) and G5 group (background/bottom, *p* < .05 as estimated by iceLogo). The size of an amino acid in the figure reflects the difference in the frequency between G1 and G5. (E) Top10 most significantly enriched sequence motifs extracted using motifeR (minimal occurrence = 20 and *p* < .000001) from the 15 A.A. sequence windows surrounding the P‐sites in G1 and G5. (F) Selected most significant results of kinase‐substrate enrichment analysis (Fisher's exact test *p* < .05). The site‐specific kinase‐substrate annotation was retrieved from OmniPath database. (G) Selected most significant results of the protein‐level annotation gene ontology biological process (GOBP) terms enrichment analysis (Fisher's exact test *p* < .05; relative to the number of phosphoproteins per category). Number of phosphorylation sites and phosphoproteins are shown in brackets. (H and I) Networks of P‐sites corresponding to the TOP most significant GOBP terms from (G) are depicted. The node fill colour indicates the MetScore; the border and node size indicate the MetScore segment.

Based on the heterogeneity of the metformin response among the 12 cell lines, we contemplated that assigning a consensus score to each P‐site quantifying its responsivity across the panel would enable the functional stratification of the P‐sites, and P‐sites perturbed by metformin in all or most of the cell lines could be curated as a CRC‐specific ‘metformin signature’. We therefore devised a scoring strategy named ‘MetScore’ (Figure 4A). For every P‐site, a ‘MetScore’ is assigned as a sum of cell lines in which a P‐site was significantly up‐regulated (+1), non‐regulated (+0) or down‐regulated (−1) using the threshold of *p* < .01 and an absolute fold change > 1.5 to determine the statistical significance. MetScore stratified all P‐sites into five segments, G1–G5 (Table S5), with G1 encompassing the mostly up‐regulated P‐sites (in at least four CRC cell lines) and G5 encompassing P‐sites mostly down‐regulated (in at least four lines). The stratification criteria of the P‐sites into the 5 segments are further illustrated in the histogram in Figure 4A. Intriguingly, the P‐sites in the G1, G4 and G5 MetScore segments showed on average significantly larger *site‐specific functional scores* ^71^ than the P‐sites in the G3 segment (i.e., the least perturbed segment). This result supports a greater cell fitness relevance of P‐sites with extreme MetScores, as compared with the unperturbed sites (Figure 4B). Remarkably, a small list of P‐sites (*n* = 43 and 12) were consistently up‐ or down‐regulated in at least 10 cell lines (|MetScore| ≥ 10; Figure 4A and Table 1). The only P‐site with a positive MetScore of ‘12’ (up‐regulated in all cell lines) was the progesterone receptor membrane component 2 (PGRMC2) Ser 104. The protein functions as a trans‐membrane progesterone receptor and has been proposed as an essential player in adipocyte glucose metabolism.^95^ According to the PhosphoSitePlus database,^65^, ^66^ the Ser 104 P‐site has been identified in multiple high‐throughput studies; however, it is not known whether it carries any regulatory function. Additionally, two P‐sites with MetScore = 11, armadillo repeat‐containing protein 10 (ARMC10) Ser 45 and stromal interaction molecule 1 (STIM1) Ser 257 have been respectively validated as modulators of AMPK mediated mitochondrial dynamics^96^ and intracellular calcium flux during exercise.^97^ On the other side, the period circadian protein homolog 2 (PER2) Ser 977 P‐site was one of the most commonly down‐regulated sites (MetScore = −11). The function of this P‐site has not been described yet while the circadian regulator PER2 has been previously shown in controlling lipid metabolism.^98^ Functional analyses for P‐sites with highest and lowest MetScores are therefore promisingly warranted in the future to understand their associations with metformin.

**TABLE 1:** P‐sites identified as the common metformin signature. Selected P‐sites with absolute MetScore ≥ 10. The fold change column indicates the averaged regulation direction and extent for each P‐site following 24‐h metformin treatment across 12 CRC cells. Reg. site ‘+’ indicates the site had a described regulatory function in the PhosphoSitePlus database

MetScore	Gene name	P‐site	Protein name	Fold change upon metformin	Reg site
12	PGRMC2	S104	Membrane‐associated progesterone receptor component 2	**20.78**	
11	ARMC10	S45	Armadillo repeat‐containing protein 10	**5.81**	+
11	ASPSCR1	S502	Tether containing UBX domain for GLUT4	**3.12**	
11	BCLAF1	S196	Bcl‐2‐associated transcription factor 1	**4.88**	
11	GPAT3	S68	Glycerol‐3‐phosphate acyltransferase 3	**8.77**	
11	PRPF4B	S8	Serine/threonine‐protein kinase PRP4 homolog	**2.93**	
11	SRRM2	T367	Serine/arginine repetitive matrix protein 2	**4.08**	
11	STIM1	S257	Stromal interaction molecule 1	**9.55**	+
10	ANXA2	S2	Annexin A2	**3.83**	
10	ANXA2	T3	Annexin A2	**3.81**	
10	ARHGEF11	S663	Rho guanine nucleotide exchange factor 11	**4.52**	
10	BAD	S99	Bcl2‐associated agonist of cell death	**2.31**	+
10	CFL1	S8	Cofilin‐1	**3.09**	
10	CLINT1	S166	Clathrin interactor 1	**4.68**	
10	EEA1	S52	Early endosome antigen 1	**7.33**	
10	EEF2	S502	Elongation factor 2	**5.24**	
10	EIF5	S10	Eukaryotic translation initiation factor 5	**3.62**	
10	FIP1L1	S492,T494	Pre‐mRNA 3′‐end‐processing factor FIP1	**3.23**	
10	HMGA1	S49	High mobility group protein HMG‐I/HMG‐Y	**3.06**	
10	HNRNPH1	S23	Heterogeneous nuclear ribonucleoprotein H	**9.21**	
10	HNRNPM	S432	Heterogeneous nuclear ribonucleoprotein M	**3.87**	
10	MAP4	S928	Microtubule‐associated protein 4	**3.82**	+
10	MAP7D1	S313	MAP7 domain‐containing protein 1	**3.97**	
10	NUFIP2	S652	Nuclear fragile X mental retardation‐interacting protein 2	**2.65**	
10	PCYT1A	S331	Choline‐phosphate cytidylyltransferase A	**3.09**	
10	PHRF1	S814	PHD and RING finger domain‐containing protein 1	**3.20**	
10	PTMA	S10	Prothymosin alpha	**2.40**	
10	RANBP1	S188	Ran‐specific GTPase‐activating protein	**3.16**	
10	RANBP2	S1955	E3 SUMO‐protein ligase RanBP2	**5.11**	
10	SAFB2	T193	Scaffold attachment factor B2	**2.03**	
10	SCAMP2	S319	Secretory carrier‐associated membrane protein 2	**11.76**	
10	SCAMP2	S320	Secretory carrier‐associated membrane protein 2	**11.12**	
10	SCRIB	S1276	Protein scribble homolog	**8.44**	
10	SLC12A2	S77	Solute carrier family 12 member 2	**8.31**	+
10	SNX27	S51	Sorting nexin‐27	**7.11**	
10	SRRM2	T359,T367	Serine/arginine repetitive matrix protein 2	**4.71**	
10	SRSF6	S119	Serine/arginine‐rich splicing factor 6	**3.16**	
10	SUGP1	S409	SURP and G‐patch domain‐containing protein 1	**3.69**	
10	TNKS1BP1	S1620	182 kDa tankyrase‐1‐binding protein	**2.47**	
10	UGDH	S476	UDP‐glucose 6‐dehydrogenase	**13.45**	
10	ZC3H13	S370,S372	Zinc finger CCCH domain‐containing protein 13	**2.49**	
10	ZC3H13	S370,Y374	Zinc finger CCCH domain‐containing protein 13	**4.68**	
10	ZC3H13	S986	Zinc finger CCCH domain‐containing protein 13	**4.46**	
−10	BAHD1	S184	Bromo adjacent homology domain‐containing 1 protein	**−4.89**	
−10	JPT2	S97	Jupiter microtubule associated homolog 2	**−4.56**	
−10	LASP1	T104	LIM and SH3 domain protein 1	**−4.62**	
−10	LMTK2	S1450	Serine/threonine‐protein kinase LMTK2	**−6.38**	+
−10	MTDH	S568	Protein LYRIC	**−2.89**	
−10	RBMX	S208	RNA‐binding motif protein, X chromosome	**−2.53**	
−10	RPRD2	S758,T763	Regulation of nuclear pre‐mRNA domain‐containing protein 2	**−3.89**	
−10	SF3B1	T426	Splicing factor 3B subunit 1	**−3.59**	
−10	STMN1	S38	Stathmin	**−5.02**	+
−10	TLE3	T334	Transducin‐like enhancer protein 3	**−4.46**	
−11	PER2	S977	Period circadian protein homolog 2	**−3.76**	
−11	ZDHHC5	S621	Palmitoyltransferase ZDHHC5	**−4.89**	

Following, we assessed known regulatory P‐sites with determined MetScores (Figure 4C). A total of 66 regulatory kinases or phosphatase P‐sites were identified and significantly enriched in G1, G2, G4 and G5 segments compared with the unperturbed P‐sites in the G3 segment (*p* = .0072, Fisher's exact test). Among them, 36 were annotated as regulating enzymatic activity by PhosphoSitePlus. In particular, we found two mTOR regulatory sites, Ser 1261 and Ser 2481. While the AMPK substrate mTOR Ser 1261^99^ was mostly up‐regulated with MetScore of ‘8’, the ‘classic’ mTOR‐activating autophosphorylation site, Ser 2481, only had a MetScore of ‘−2’ (Figure S8). Thus, Ser 2481 appeared to be only weakly modulated by metformin in most CRC cell lines – this observation agrees well with the result in MCF7 cells,^39^ and therefore reinforces one of the conclusions of Sacco et al. that PI3K‐mTOR pathway was rewired rather than completely turned off upon 24‐h metformin treatment in cancer cells.^39^

Next, we performed a series of functional enrichment analyses for G1 and G5 segments. *First*, we compared the amino acid sequences surrounding the G1 and G5 sites using iceLogo (*t*‐test *p* < .05; Figure 4D),^60^ revealing that basic amino acids (Arg and Lys) at the positions preceding the P‐sites and acidic amino acids (Glu and Asp) following the P‐sites were enriched in the G1 segment. Importantly, the G1 sites directly emerged an optimal AMPK motif^100^ with a strong preference for basic residues at −4 and −3 positions and hydrophobic residues at −5 and +4 positions relative to the P‐site. On the other hand, there was a relative enrichment of proline at +1 position and basic amino acids (Arg and Lys) at the positions following a P‐site in G5, indicating metformin tended to preferentially down‐regulate proline‐directed protein kinases such as MAPKs and cyclin‐dependent kinases (CDKs). Reassuringly, the motif enrichment results by motifeR (Figure 4E)^61^ showed that all top 10 most significant motifs in G5 have a proline at +1 position (Figure 4E), including the S/T‐P‐x‐K/R, the classic substrate motif for CDKs,^101^ as well as P‐x‐S/T‐P, the established motif for MAPKs.^102^ In contrast, the AKT substrate motif, R‐x‐R‐x‐x‐S/T,^103^ was enriched in G1 sites. *Second*, we performed an enrichment analysis for known kinase targets extracted from the OmniPath database^68^, ^69^, ^104^ (Fisher's exact test *p* < .05; Figure 4F). In addition to CDKs enriched in G5, this analysis revealed mTOR substrates were enriched in G5, while the AMPK (PRKAA1) substrates were enriched in G1, confirming the importance of AMPK‐mTOR axis following metformin treatment. The G1 segment also enriched substrates of kinases previously reported to be activated by metformin,^36^, ^39^ such as AKT Serine/Threonine Kinase 1 (AKT1), MAPK Activated Protein Kinase 2 (MAPKAPK2; MK2) or protein kinase D (PRDKD1; PKD). *Third*, we analysed significant GO BPs in G1 and G5 segments at the P‐site level. We identified terms related to mTOR signaling, autophagy regulation, cell proliferation and general cellular processes such as gene expression, mRNA splicing and protein modifications; many are overlapping with Figure 2E (i.e., the protein ID based enrichment), as expected (Fisher's exact test *p* < .05; Figure 4G). As particular examples, most P‐sites in proteins involved in mTOR signaling were down‐regulated whereas the autophagosome maturation process contained predominantly up‐regulated sites (Figures 4H and I). We additionally identified a significant overrepresentation of the Wnt signaling pathway in G5 (Figure 4G), which has been shown to be hyperactivated in almost all CRCs as an initiating event.^105^ Hypophosphorylation in Wnt pathway might indicate a potential anti‐CRC mechanism of metformin. *Last*, we observed a significant enrichment (Fisher's exact test *p* = .014; Figure S11) of P‐sites corresponding to metformin treatment‐associated phosphoproteins identified in an in vivo mouse liver dataset^36^ in the G1 segment, suggesting that some of the G1 sites might be universally phosphorylated as a response to metformin in diverse experimental models.

To summarise, our multi‐cell line data in CRC were used to assign a unique MetScore per each P‐site in the phosphoproteome, which facilitated the P‐site specific functional analysis towards the in‐depth understanding of metformin signaling.

### 3.6 Inferring the kinase activity landscape and signaling nodes in CRC cells following metformin treatment

**FIGURE 5:**
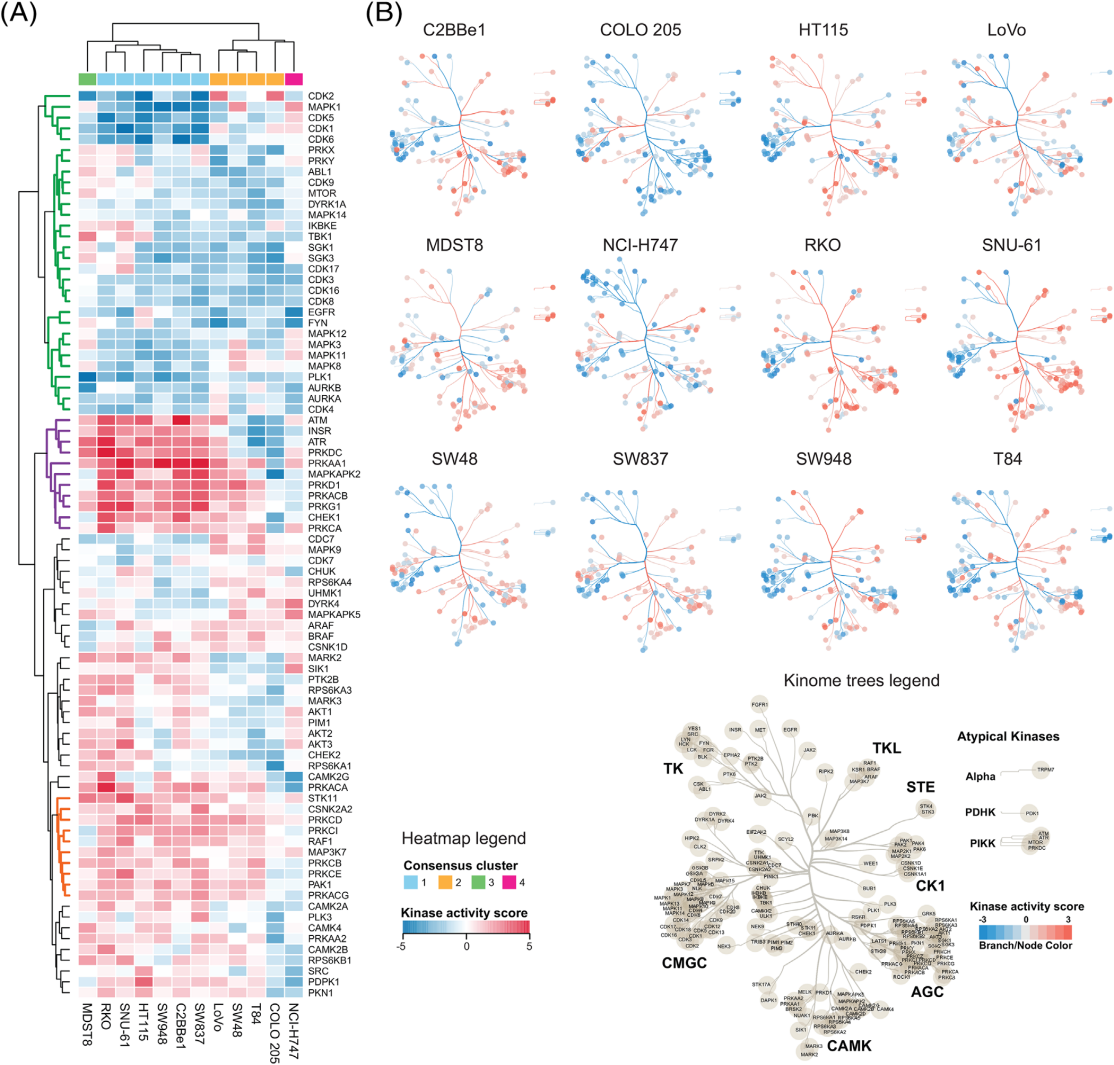
Kinase activity landscape highlights the heterogeneity of metformin response across the cell lines. (A) Hierarchical clustering analysis of kinase activity scores estimated using decoupleR rewired after 24 h of metformin treatment. Only kinases with statistically significant activity scores (*p* < .05) in at least one cell line and estimated activities in all 12 cell lines are shown. (B) The metformin‐rewired kinomes were visualised per cell line. The branch and node colour mapping correspond to the kinase activity score. Only those nodes and branches with a valid score per cell line are shown. The legend shows all branches and kinases with computed activity scores in at least one cell line.

To infer the kinase activity response per cell line (Table S6), we performed an enrichment on the phosphoproteomic data using decoupleR,^74^ leveraging the kinase‐P‐site interactions from the OmniPath database.^68^, ^69^ The normalized enrichment scores (NESs) estimated by decoupleR for each kinase in every cell line were filtered (significantly regulated in at least one of the cell lines, decoupleR *p* < .05) and the NESs were subjected to HCA, revealing several profile clusters for kinases (Figure 5A). The group of kinases that showed mostly down‐regulated activities across the cell lines (Figure 5A, green cluster) includes many kinases regulating cellular growth and proliferation, such as mTOR, CDKs, mitotic regulators AURKA, AURKB, PLK1 and MAPKs, but also several tyrosine kinases such as EGFR, FYN and ABL1 and a dual specificity kinase DYRK1A, which is not evident from the above P‐site level analyses. On the other hand, the kinase group up‐regulated in the consensus cluster 1 cells (i.e., RKO, SNU‐61, HT115, SW948, C2BBe1, SW48; Figure 5A, purple cluster; Figure 5A) harbours AMPK (PRKAA1) as well as other metabolic regulators such as the insulin receptor (INSR), a tyrosine kinase regulating glucose metabolism. Remarkably, this cell line cluster also showed activity increase for the upstream CDKs regulators in response to different stress stimuli, such as ATM, ATR, the catalytic subunit of DNA‐PK (PRKDC), and CHEK1, which potentially explains a stronger down‐regulation of CDKs in these cell lines. Serine/threonine‐protein kinase STK11 (STK11; LKB1), a kinase essential for AMPK activation under low ATP conditions^31^ was determined to be activated in most of the cell lines (Figure 5A, orange cluster). The cell line‐specific, metformin‐induced kinase activity landscape is further illustrated by the kinome trees (Figure 5B). The colour mapping of branches and nodes corresponding to the kinase activity scores highlights variable fingerprints of cell signaling states, for example, in the CAMK and AGC kinome branches. Surprisingly, in the COLO 205 cell line, the reduced activity was inferred for most kinases, while the global preference for P‐site down‐regulation was not observed in COLO 205 (see the volcano plot in Figure 2B). This discrepancy supports the value of kinase activity inference to analyse the phosphoproteomic data.

**FIGURE 6:**
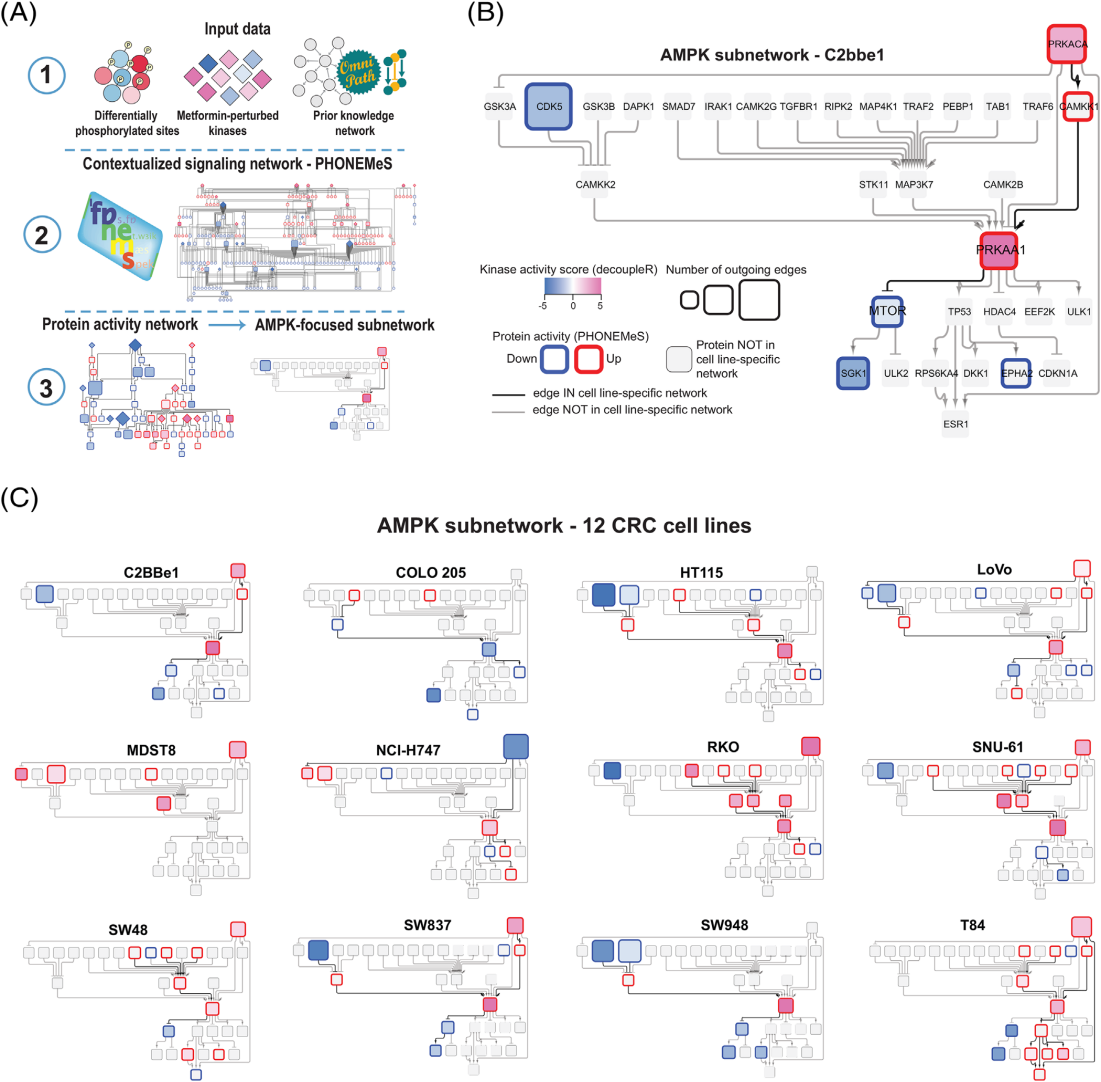
Network analysis using PHONEMeS reveals a substantial heterogeneity in the AMPK‐focused subnetwork across CRC cells. (A) PHONEMeS analysis workflow. The results of the differential analysis (*t*‐value; limma), the results of the kinase activity analysis (normalised kinase activity score, decoupleR) and prior knowledge network containing protein–protein and kinase–P‐sites interactions retrieved from OmniPath were used as an input for PHONEMEeS to reconstruct cell line‐specific signalling networks. A general AMPK‐focused subnetwork was constructed using all two‐step up‐ and down‐stream neighbours of AMPK included in the network in at least one of the cell lines. The cell line‐specific nodes and edges were highlighted. (B) An example of C2BBe1 AMPK‐focused subnetwork. The border colour corresponds to the protein activity scores calculated using PHONEMeS; the fill colour mapping illustrated the decoupleR kinase activity score. The bold edges are part of the C2BBe1 subnetwork. (C) Subnetworks from all 12 CRC cell lines illustrate the cell‐specificity of metformin‐induced signalling. The position of the nodes (proteins) and edges is the same as in (B).

To derive mechanistic insights into MoA of metformin in individual cell lines, we contextualised the networks using PHONEMeS.^76^, ^106^ Three inputs were used for this analysis (Figure 6A), (i) the top 15% of differentially expressed P‐sites, (ii) the top 15% of differentially activated kinases and (iii) the PKN extracted from OmniPath database consisting of protein‐protein and kinase‐P‐site interactions.^68^, ^69^ Using these inputs, PHONEMeS reconstructed a coherent path connecting deregulated kinases with deregulated P‐sites resulting in large signaling networks. The resulting protein activity networks contained perturbed kinases (from the inputs) as well as the inferred intermediate nodes and a computed score reflecting the activity of each node (Figures S12A–L). As an example of prominent modules, we focused the subnetwork on AMPK and its closest upstream regulators and downstream effectors across cell lines (Figures 6B and C). The reconstructed subnetworks illustrated the substantial differences between the cell lines in their response to metformin after 24 h (Figure 6C). *First*, AMPK, as the central node, was not perturbed the same across cell lines. In MDST8, AMPK was not activated. In COLO 205, the outlier in the kinome visualisation (Figure 5B), AMPK activity was inferred to be down‐regulated. The rest of the cell lines showed AMPK activation, but with a different extent. *Second*, the upstream signals leading to AMPK activation seem to be divergent between cells. Previously, AMPK activity was reported to be regulated by several kinases in a context‐dependent manner.^107^ For example, in the context of low energy (ATP) conditions, STK11 is essential for AMPK phosphorylation at Thr 172 and AMPK activation.^31^ Under conditions of calcium flux, however, despite the same residue Thr 172 is phosphorylated, AMPK activity is regulated by the calcium‐sensitive kinase calcium/calmodulin‐dependent protein kinase kinase 2/beta (CAMKK2).^32^, ^33^, ^34^ Furthermore, mitogen‐activated protein kinase kinase kinase 7 (MAP3K7; TAK1) controls AMPK during lysosomal injury by various agents including metformin treatment.^35^ Our reconstructed signaling networks suggested STK11 was upstream of AMPK in three cell lines, while the CAMKK1 and CAMKK2 kinases were acting upstream in nine cell lines. Meanwhile, in five cell lines the MAP3K7 was found to be the upstream AMPK regulator (Figure 6C). These results largely delineated the context‐dependent AMPK activation. *Third*, the activity of the downstream AMPK nodes also showed a considerable heterogeneity. Half of the cell lines showed mTOR inactivation followed by inhibition of autophagy inhibitor serine/threonine‐protein kinase Sgk1 (SGK1)^108^ as the best path explaining the signaling outcome in four cell lines (Figure 6C). Other downstream effectors were usually not shared by more than two cell lines.

Altogether, using phosphoproteomics‐tailored bioinformatic tools, the metformin‐signaling leading to AMPK activation and downstream signal propagation was revealed to be orchestrated by different kinase activities and dependent on specific cellular context.

### 3.7 Leveraging the metformin signature to predict novel metformin–drug interactions

**FIGURE 7:**
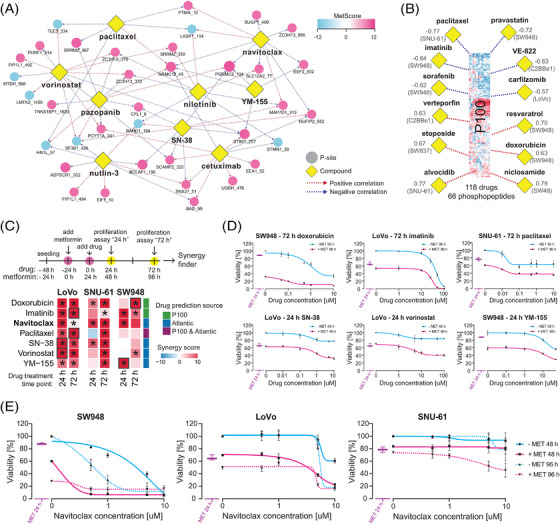
Identification of CRC‐specific metformin–drug interactions. (A) P‐site–drug network was reconstructed using significant associations of the metformin signature P‐sites (absolute MetScore ≥10) retrieved from ATLANTiC database. The drugs/compounds are highlighted by yellow diamonds; the P‐sites are shown as circles. The colour of the circles indicates the MetScore. The colour of the edges indicates whether the abundance of a P‐site was shown to be significantly positively or negatively correlated with a sensitivity to a drug in the ATLANTiC database. (B) Visualisation of the six selected most correlating (red dashed line) and anticorrelating (blue dashed line) drugs based on the P100 correlation analysis. The numbers indicate the strongest correlation score within the cell line panel estimated for a cell line indicated in brackets. (C) The scheme summarises the experimental design of the drug combination experiments. The heatmap shows the 75th percentile Loewe synergy score from the drug–metformin concentration range, and the asterisks indicate statistical significance (*p* < .05) of the combined effect across the metformin–drug concentration range. The analysis was performed using the SynergyFinder R package.^87^ The black rectangles highlight results visualised in (D). (D) Representative plots showing the effects of a metformin–drug combinations on cellular viability. An example for each one of the drugs was selected based on the max synergy score as depicted in (C). (E) Synergistic effects of navitoclax in all three cell lines. (D and E) ‘MET 24 h’ indicates the viability of a cell line after 24 h of metformin treatment – before the second drug was added to the media. The data were expressed as percentage of viability of the average untreated control per drug combination.

Recently, several computational tools and resources have enabled annotating specific P‐site as a known drug target^67^ or correlating the P‐site abundance^79^, ^80^, ^81^ with drug sensitivity across cell line panels.^77^ Furthermore, metformin has shown synergistic effects with CRC genotoxic chemotherapy in vitro.^18^, ^20^, ^21^, ^109^ Therefore, we asked whether we could leverage the metformin phosphoproteomic signatures we discovered to predict the metformin–drug interactions in CRC. We first queried the 55 P‐sites with a |MetScore| ≥ 10 (Figure 4A and Table 1) in the ATLANTiC website,^77^ which hosts significant drug‐sites association predicted based on a larger CRC panel of 65 cell lines (hereafter, CRC65). From these drug‐sites associations, we extracted nine compounds with different targets and MoA significantly associated with the most P‐sites (Figure 7A). In a second strategy, we took advantage of the P100 dataset^79^, ^80^, ^81^ and performed a correlation analysis of our metformin phosphoproteomic profiles with those of 118 drugs (Figure S13A), with the purpose of identifying drugs strongly correlating or anticorrelating with metformin‐induced phosphoproteome change based on 96 P‐sites (see prominent examples in Figure 7B). Importantly, many of the predicted compounds are US FDA approved cancer drugs (carfilzomib, cetuximab, doxorubicin, etoposide, imatinib, paclitaxel, pazopanib, niclosamide, nilotinib, sorafenib and vorinostat) or promising compounds evaluated in clinical trials for cancer indications (alvocodib, pravastatin, navitoclax, niclosamide, SN‐38, verteporfin, VE‐822 and YM‐155). A few drugs, such as paclitaxel or vorinostat, were further annotated in PTMsigDB^67^ (Figure S13B). Intriguingly, according to a literature investigation, many drugs in this list have been shown to interact with metformin in in vitro or in vivo models of several cancer types (summarised in Table S7).

Next, we selected 3 drugs (i.e., doxorubicin, imatinib and paclitaxel) based on the P100 signature correlation analysis and 5 drugs (i.e., navitoclax, paclitaxel, SN‐38, vorinostat and YM‐155) based on the ATLANTiC associations (Figure 7C) for confirmative proliferation assays in LoVo, SNU‐61 and SW948 cells. This confirmative experiment was designed to follow the rationale of the phosphoproteomic investigation above, with a 24‐h ‘nutrient exhaustion’ and another 24‐h metformin treatment before the addition of drugs. This time period resulted in 10–30% inhibition of proliferation by 10 mM metformin in these cell lines (Figure S14D). The combined effects were validated at 24 and 72 h (Figure 7C). As a result, based on SynergyFinder,^87^ significant synergistic effect of all drugs were confirmed in at least one cell line and timepoint (Figures 7C–E). Notably, the highest synergy came from the combination of metformin with navitoclax (ABT‐263), a BCL‐2/BCL‐xL inhibitor (Figure 7E). The maximum LOEWE synergy scores and experiment‐wide *p* values were 52.45 (*p* < 2.20 × 10E−16), 31.07 (*p* = 3.02 × 10E−07) and 20.57 (*p* = 9.81 × 10E−4) for SW‐948 (24 h), LoVo (24 h) and SNU‐61 (72 h), respectively, indicating a strong synergetic effect of navitoclax with metformin.

In conclusion, leveraging our P‐site specific MetScore and available drug–phosphoproteomics resources enabled identification of drugs potentially interacting with metformin.

### 3.8 A website hosting the data basis for metformin induced regulation in CRC cells

In addition to supplementary tables, to facilitate the exploration of the metformin proteomic and phosphoproteomic profiles, we presented our entire data resource as a website (https://yslproteomics.shinyapps.io/Metformin/). This website supports interactive visualisations such as the distribution plots of total protein and phosphopeptide abundances per CRC cell line, the fold‐change bar plots of each P‐site following metformin treatment, and HCA‐based heatmaps of the regulatory profiles of user‐specified proteins and P‐sites. Furthermore, this website hosts extended versions of Tables S2 and S3 containing all quantitative information and the results of the statistical analysis.

## 4 DISCUSSION

Type II diabetes patients have been shown to suffer from increased risk of developing CRC.^24^ Mounting evidence suggests that the first line T2D drug, metformin, could be beneficial in preventing the CRC development and improving the cancer prognosis.^24^ The MoA of metformin is pleiotropic, affecting both whole organism and cellular levels,^23^, ^37^ but not yet completely understood. Moreover, large‐scale proteomic and phosphoproteomic studies have indicated that metformin does not affect a limited set of individual targets, but rather modulates the activity of multiple proteins and kinases resulting in an extensively rewired signaling network.^39^ Due to the complexity of the metformin‐rewired signaling network and the individual genomic variability, phosphoproteomics is a compelling analytical approach due to its ability to quantify thousands of P‐sites across cell line panels. Additionally, recent technological, experimental and computational advances have improved the depth and breadth of phosphoproteomic analysis and the data interpretation.

Here we performed a deep proteomic and phosphoproteomic analysis (Figure 1) of metformin‐treated molecularly heterogenous CRC cell lines^56^ after different timepoints to elucidate the MoA of metformin in CRC and address the heterogeneity of drug response in a panel with a variable baseline proteome and phosphoproteome. Because the cancer cell culturing system is nutrient‐rich, following a published investigation in MCF7 cells,^39^ we cultured the CRC cells for 24 h without refreshing the media to achieve a partial ‘nutrient exhaustion’, and applied 10 mM metformin, which appears at a much higher concentration than clinically used. Such a condition was essential to observe AMPK activation in cancer cells.^39^ It should be stressed that, upon the metformin concentration of 10 mM, the total proteome changes were minimal (i.e., <2%) across all 12 cell lines after the 24‐h treatment; and two cell lines (MDST8 and NCI‐H747) only showed a very small extent (i.e., 0.8 and 4.1%) of regulation even at the phosphoproteome level. These results, together with many in vitro studies of metformin using a similar or higher metformin concentration (Table S7), suggest the trade‐offs between high metformin concentrations and detectable protein‐level regulations in cultured cancer cells.

We confirmed the previous observation reported in a breast cancer cell line that metformin remodelled the phosphoproteome within a 24‐h time window.^39^ We also observed phosphorylation up‐regulation for metformin‐interacting lysosomal proteins,^70^ which warrants future mechanistic investigation. Furthermore, we observed that the 24‐h response was highly heterogenous at multiple levels, as demonstrated specifically via (1) distinctive response at the level of individual differentially phosphorylated P‐sites and motifs (Figure 2), (2) differential regulation of phosphorylation event occurring within specific biological processes (Figure 2), (3) differential kinase activity regulation (Figure 5) and (4) reconstructed signaling networks (Figure 6). These findings highlighted that the drug‐induced signaling can be highly context‐dependent in CRC and emphasised the importance of using multiple cell lines to study in vitro cancer cell signaling. The favourable reproducibility of Phos‐DIA is fundamental to derive these observations in individual cell lines.

On the other hand, tens of phosphorylation sites and a set of protein kinases and biological processes were commonly regulated in most cell lines (Figure 4 and Table 1). The identification of these phosphorylation events was facilitated by assigning each P‐site a MetScore reflecting the robustness of a P‐site regulation within the panel of CRC cell lines. We identified 55 most consistent P‐sites regulated in at least 10 CRC cell lines, which can be considered as the common ‘metformin signature’ for CRC cells. The identification of a robust metformin signature might have clinical implications. *First*, in CRC patients with type II diabetes, metformin usage daily may lead to profound effects on the phosphoproteome of the tumour cells. The metformin signature P‐sites might serve as a resource to develop potential assays for monitoring metformin sensitivity and intended efficacy in cancer patients. *Second*, through regulation of specific P‐sites, metformin might prime the cancer cells to be more sensitive or more resistant to conventional genotoxic chemotherapeutic agents or targeted cancer drugs (see below). Thus, the determination of how the phosphorylation signaling is rewired by metformin in cancer cells might be useful in optimising cancer therapeutics. In the light of the results above, it is essential to reconcile our discoveries of cell–cell heterogeneity and common P‐site regulation upon metformin treatment for the potential clinical applications. While the common P‐sites are suggestive of potential actionable therapeutic targets that are more general in CRC, the multilayered heterogeneity of metformin response highlights the importance of patient‐tailored precision medicine. This means, to ultimately optimise the cancer therapeutics expected from metformin usage, it might be helpful to measure, for example, the patient derived cells or other models regarding their personalised phosphoproteomic profiles and metformin response, using our dataset as a valuable reference.

Metformin is an attractive repurposing drug^22^ due to its low cost and a compelling safety profile. Combinations of metformin with several genotoxic CRC chemotherapy drugs have been already reported.^18^, ^20^, ^21^, ^109^ It would be ideal if metformin could either sensitise cancer cells to a drug leading to increased efficacy of the treatment, revert existing resistance or decrease the deleterious side effects of chemotherapy. Since the synergies between drugs are rather rare and tend to be cell signaling‐dependent,^110^ we leveraged the identification of the metformin signature across the cell lines and queried established P‐site abundance–drug sensitivity correlations.^77^ The rationale behind this approach relied on the assumption that if one or more P‐site abundances are positively correlated with the drug sensitivity within the CRC65 panel (i.e., higher P‐site abundance–lower IC50), the up‐regulation of the P‐site(s) might further sensitise the drug response and vice versa. The metformin induced deregulation on the same P‐sites(s) is thus prone to affect the certain drug response. Additionally, we correlated the metformin phosphoproteomic profiles of CRC cell lines with drug perturbation profiles using the P100 approach.^79^, ^80^, ^81^ Our preliminary results are promising regarding drug repurposing because most of the potential compounds identified have already been US FDA‐approved for other cancer indications (carfilzomib, cetuximab, doxorubicin, etoposide, imatinib, paclitaxel, pazopanib, niclosamide, nilotinib, sorafenib and vorinostat) or in the late stages of clinical research development for cancer treatment (alvocodib, pravastatin, navitoclax, niclosamide, SN‐38, verteporfin, VE‐822 and YM‐155); and for doxorubicin, etoposide, imatinib, navitoclax, nilotinib, nutlin‐3, paclitaxel, pazopanib, resveratrol, SN‐38, sorafenib and vorinostat, their synergy with metformin have been already reported in concrete literature in other cancer models (Table S7). Our drug predictions are thus largely consistent with the existing experimental results. Moreover, we performed in vitro experiments for 7 selected drugs predicted using different strategies and confirmed their interactions with metformin in the context of CRC. The most significant synergism was found for navitoclax, which acts as a pro‐apoptotic anti‐cancer drug by inhibiting the anti‐apoptotic proteins Bcl‐2 and Bcl‐XL. A possible mechanism of the synergism might lie in the sensitising effect of metformin towards apoptotic agents reported previously by disrupting the balance between pro‐ and anti‐apoptosis signaling^39^ or by decreasing the protein level of the Bcl‐2 family proteins.^111^, ^112^ However, uncovering the exact molecular mechanism of navitoclax∼metformin interaction in CRC warrants future pre‐clinical and clinical investigation. The fact that navitoclax has been shown to enhance sensitivity of paediatric glioma to metformin^113^ or in several p53‐defective cells previously^114^ reinforces a promising role of metformin in sensitising navitoclax effect in cancer treatment. Furthermore, due to the lack and clinical importance of such knowledge, similar large‐scale studies in the future should be designed and performed to identify drugs for which metformin would serve as an antagonist.

In conclusion, our study reveals novel and fundamental insights for understanding metformin‐induced phosphorylation signaling, including the regulation timing and extent, the cell line dependency and the signaling network and kinase landscape as the consequences of the regulation, providing a deep and substantial phosphoproteomic resource sharpening our views on metformin and its clinical potentials.

## CONFLICT OF INTEREST

J. S. R. has received funding from GSK and Sanofi and fees from Astex and Travere Therapeutics. Other authors declare no conflict of interest.

## Supporting information

Supporting Information

Supporting Information

Supporting Information

Supporting Information

Supporting Information

Supporting Information

Supporting Information
