# Supplementary material for: Phosphoproteomic analysis of metformin signaling in colorectal cancer cells elucidates mechanism of action and potential therapeutic opportunities
Source: Clin Transl Med. 2023 Feb 13;13(2):e1179. doi: 10.1002/ctm2.1179 (PMC9925373; doi:10.1002/ctm2.1179)
Supplement: Supplementary file 1 — Supporting Information [file CTM2-13-e1179-s006.docx]

Supplementary Materials for

**Phosphoproteomic Analysis of Metformin Signaling in Colorectal Cancer Cells Elucidates Mechanism of Action and Potential Therapeutic Opportunities**

Barbora Salovska *et al.*

Corresponding author email: [yansheng.liu@yale.edu](mailto:yansheng.liu@yale.edu)

**This PDF file includes:**

Supplementary figures and their captions

Figures S1-S14 (Figure S12 is provided separately as a supplementary pdf)

Supplementary tables and their captions

Table S1

Table S7

## Supplementary figures


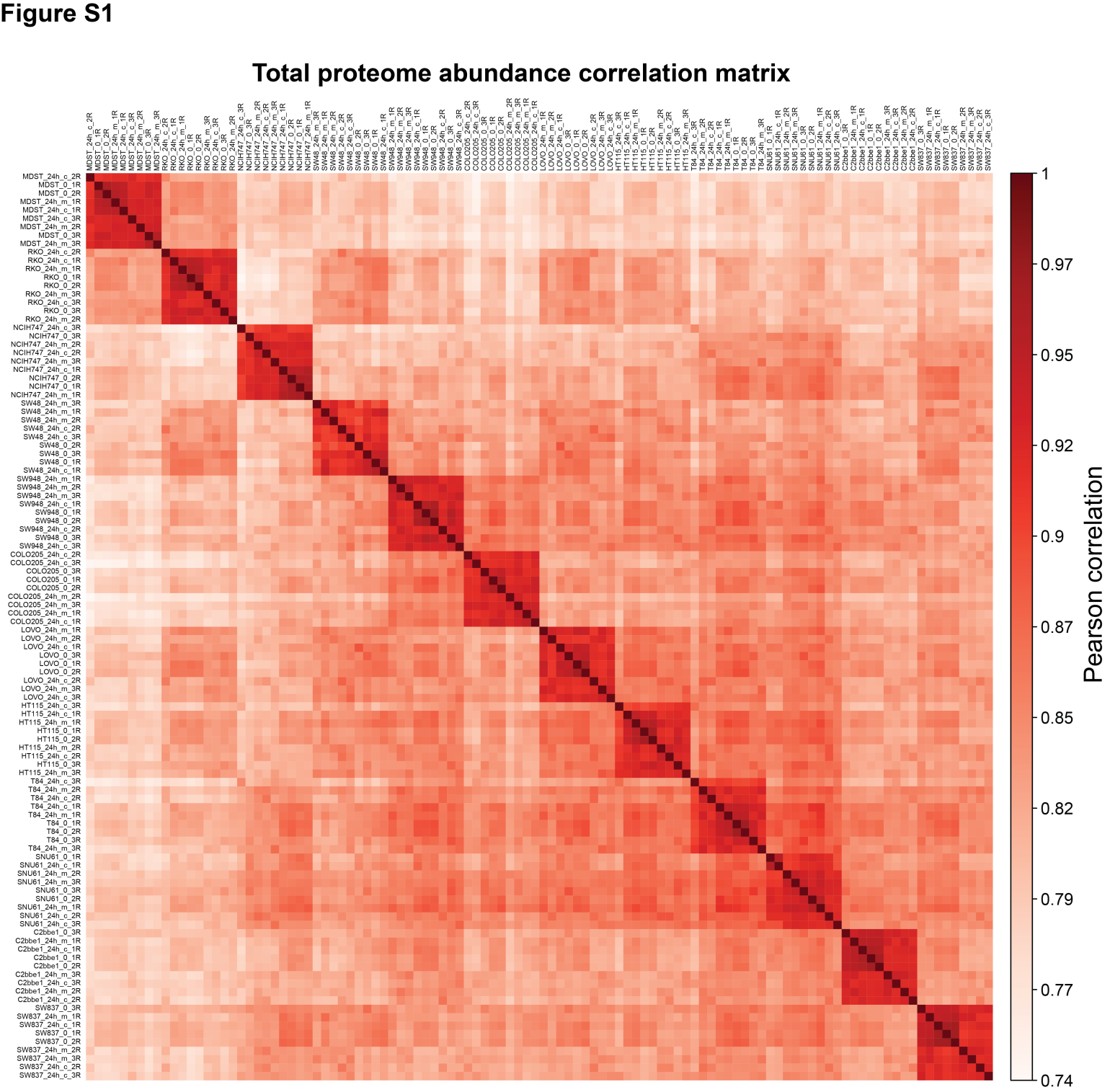


**Figure S1: Correlation analysis of the total proteome data (related to Figure 1).** Pearson correlation between samples was calculated using log2-transformed protein abundances. The heatmap shows hierarchical clustering of the correlation coefficients between samples.


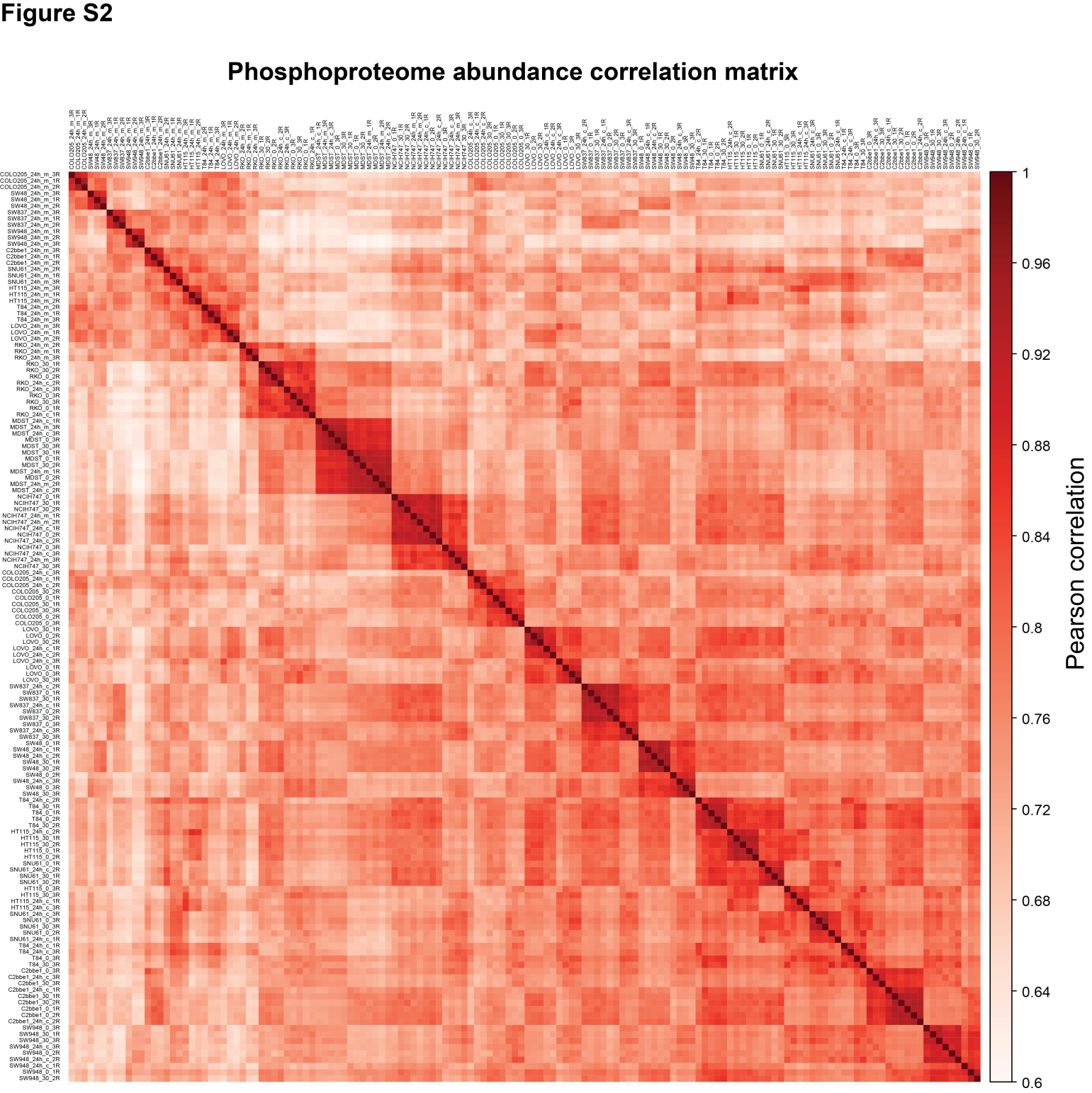


**Figure S2:** **Correlation analysis of the phosphoproteome data (related to Figure 1).** Pearson correlation between samples was calculated using log2-transformed phosphopeptide abundances. The heatmap shows hierarchical clustering of the correlation coefficients between samples.

**
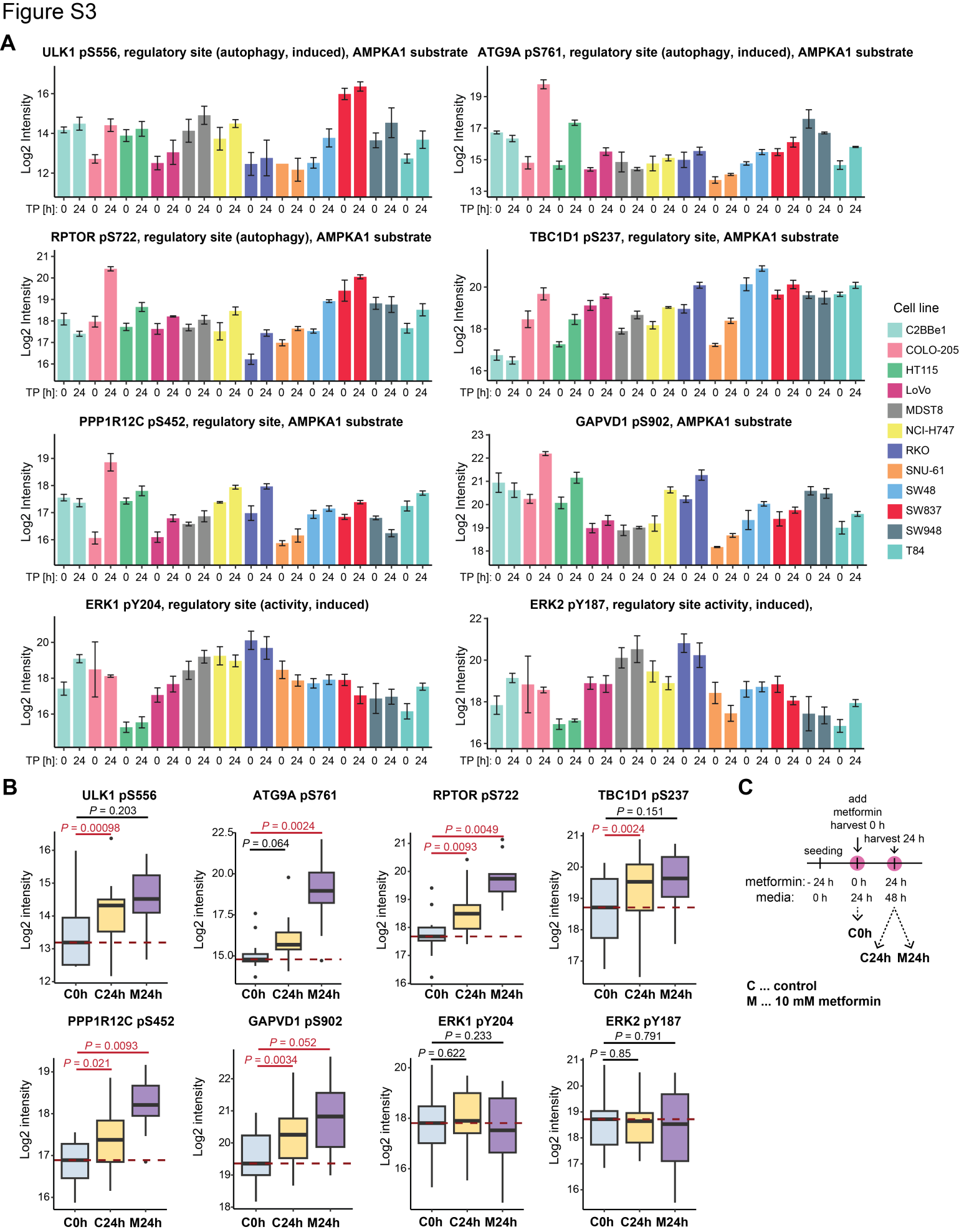
**

**Figure S3**: **Cellular response to “partial nutrient exhaustion” in untreated control samples (related to** **Figure 1**). **(A)** Phosphorylation of selected P-sites that are known AMPK substrates and regulatory ERK1 and ERK2 P-sites in control untreated samples after 24 hours (TP 0 h) and 48 hours (TP 24 h) of culturing the cells without refreshing the cell culture media. **(B)** The boxplots summarize the intensities of the P-sites in panel (A) across the 12 cell lines (n = 12) in control 0 h (C0h), control 24 h (C24h), and metformin 24 h (M24h) samples. Statistical analysis was performed using paired Wilcox test. **(C)** Depiction of the experimental design and samples visualized in this figure.


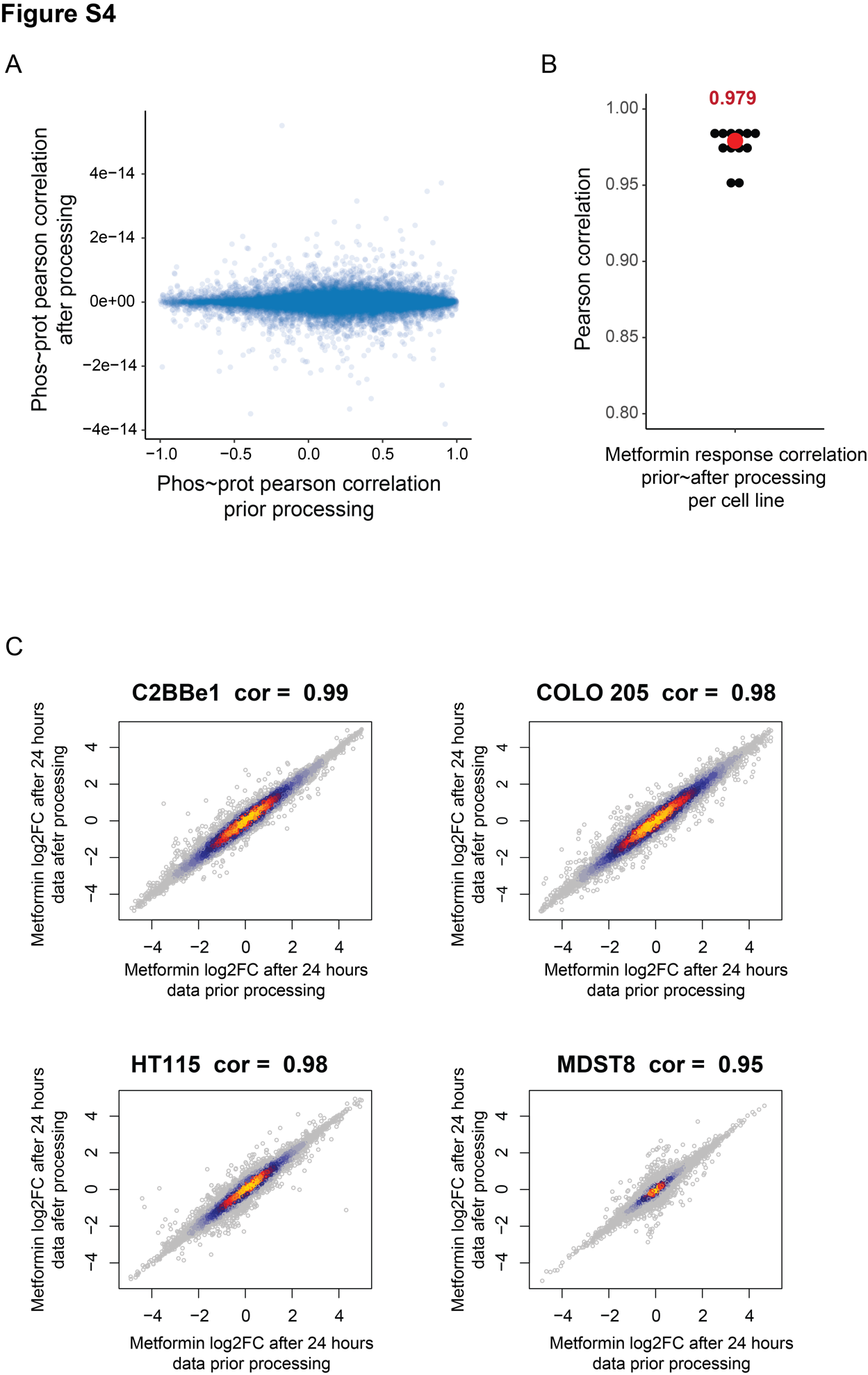


**Figure S4: Phosphopeptide level change was not driven by protein abundance change after 24 hours of metformin treatment (related to Figure 2).** **(A)** The relative protein abundances were regressed out from the respective relative phosphopeptide abundance values to detect net phosphorylation changes using linear regression (see **Methods**). The scatter plot shows the phosphopeptide-specific Pearson correlation of the phosphopeptide abundance and abundance of the corresponding total protein (phos~prot correlation) before (x axis) and after (y axis) the regression. **(B)** Pearson correlation coefficients of the log2 metformin/control fold change prior and after the regression. Each dot represents a value of an individual cell line. The red dot represents the median value. **(C)** Examples of the log2 metformin/control fold change prior and after the regression in four cell lines.


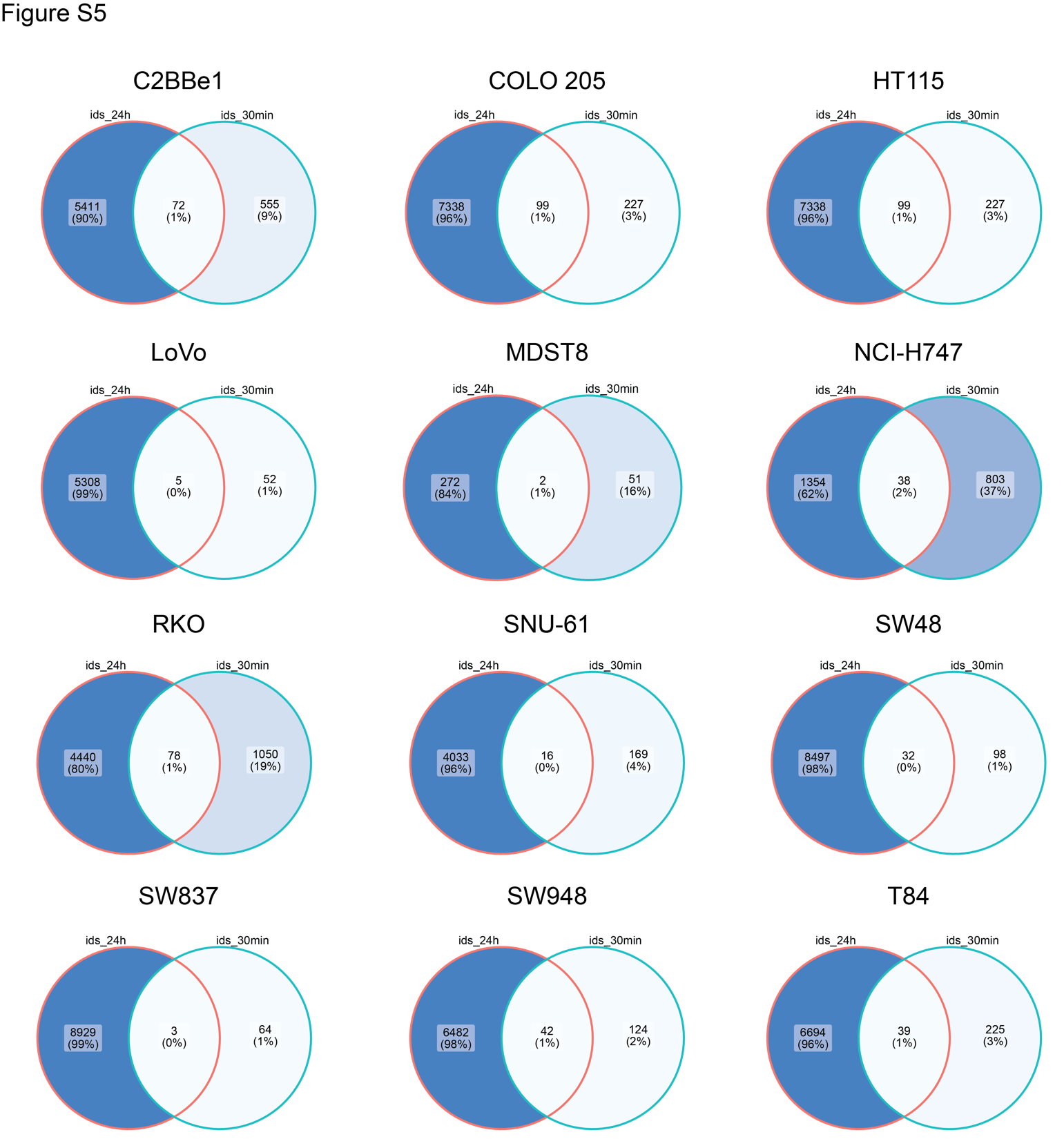


**Figure S5**: **Minor overlap of the P-sites significantly affected by the “acute” and “late” metformin treatment (related to** **Figure 2**). Venn diagrams show the overlaps between the significant P-sites (t-test *p* < 0.01, |FC| > 1.5) after 30 min and 24 h treatment with metformin for the 12 cell lines.


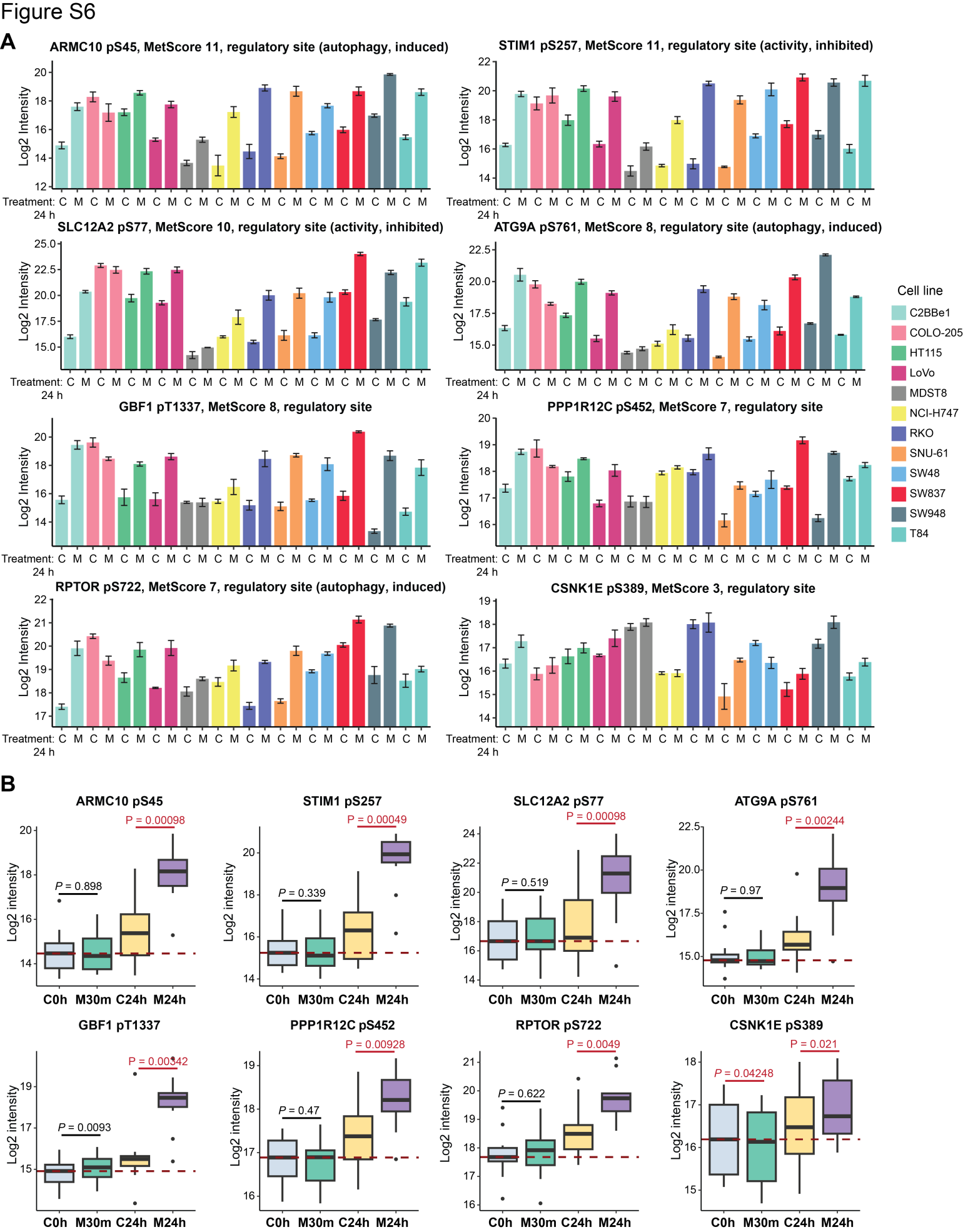


**Figure S6**: **Regulation of known AMPK substrate P-sites by metformin (related to** **Figure 2**). **(A)** Phosphorylation of selected known P-sites that are known AMPK targets based on the PhosphositePlus database after 24 hours in untreated control samples (C) or metformin-treated samples (M). **(B)** The boxplots summarize the intensities of the P-sites in panel (A) across the 12 cell lines (n = 12) in control 0 h (C0h), control 24 h (C24h), metformin 30 min (M30m), and metformin 24 h (M24h) samples. Statistical analysis was performed using paired Wilcox test.


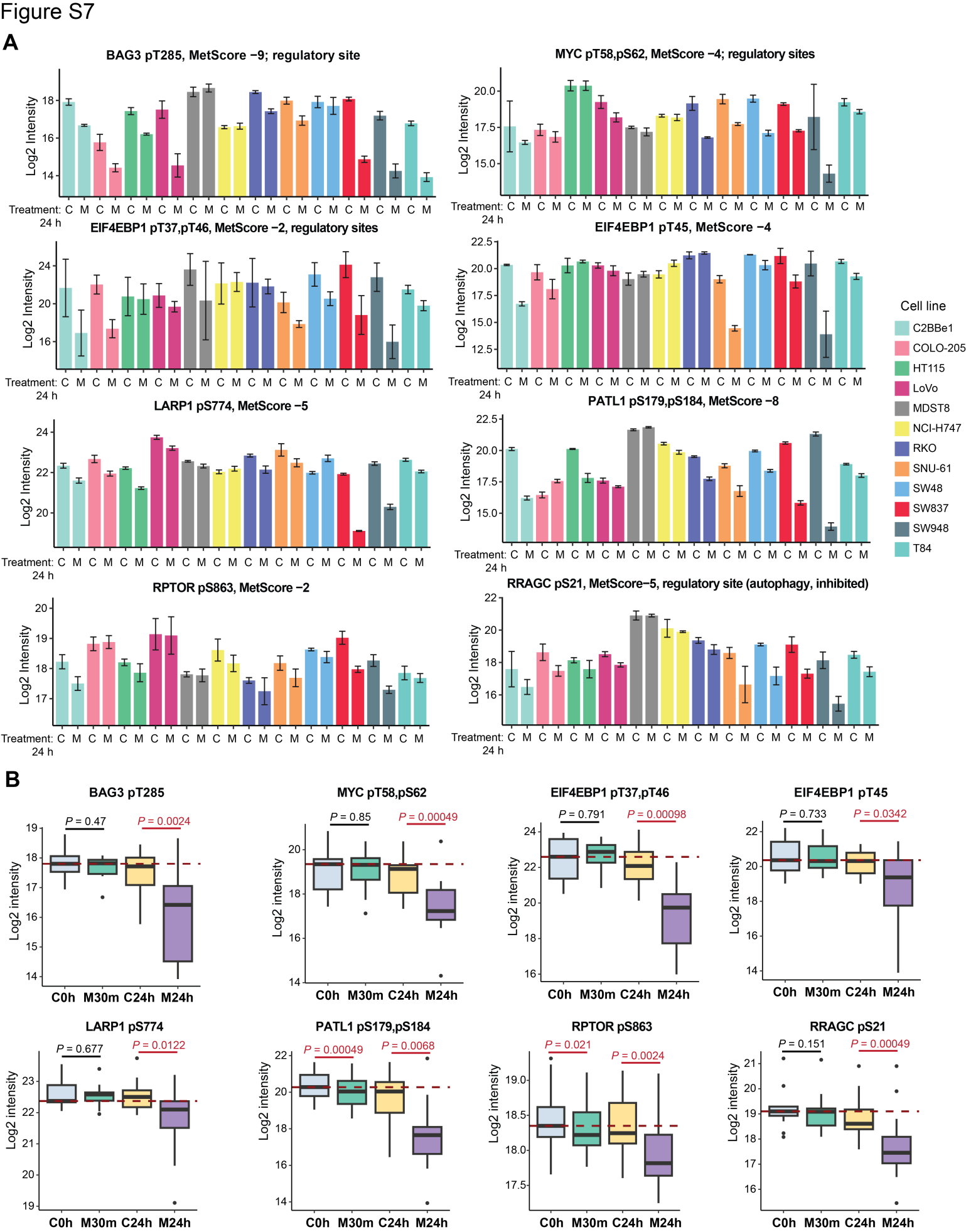


**Figure S7**: **Regulation of known mTOR substrate P-sites by metformin (related to** **Figure 2**). **(A)** Phosphorylation of selected known P-sites that are known mTOR targets based on the PhosphositePlus database after 24 hours in untreated control samples (C) or metformin-treated samples (M). **(B)** The boxplots summarize the intensities of the P-sites in panel (A) across the 12 cell lines (n = 12) in control 0 h (C0h), control 24 h (C24h), metformin 30 min (M30m), and metformin 24 h (M24h) samples. Statistical analysis was performed using paired Wilcox test.


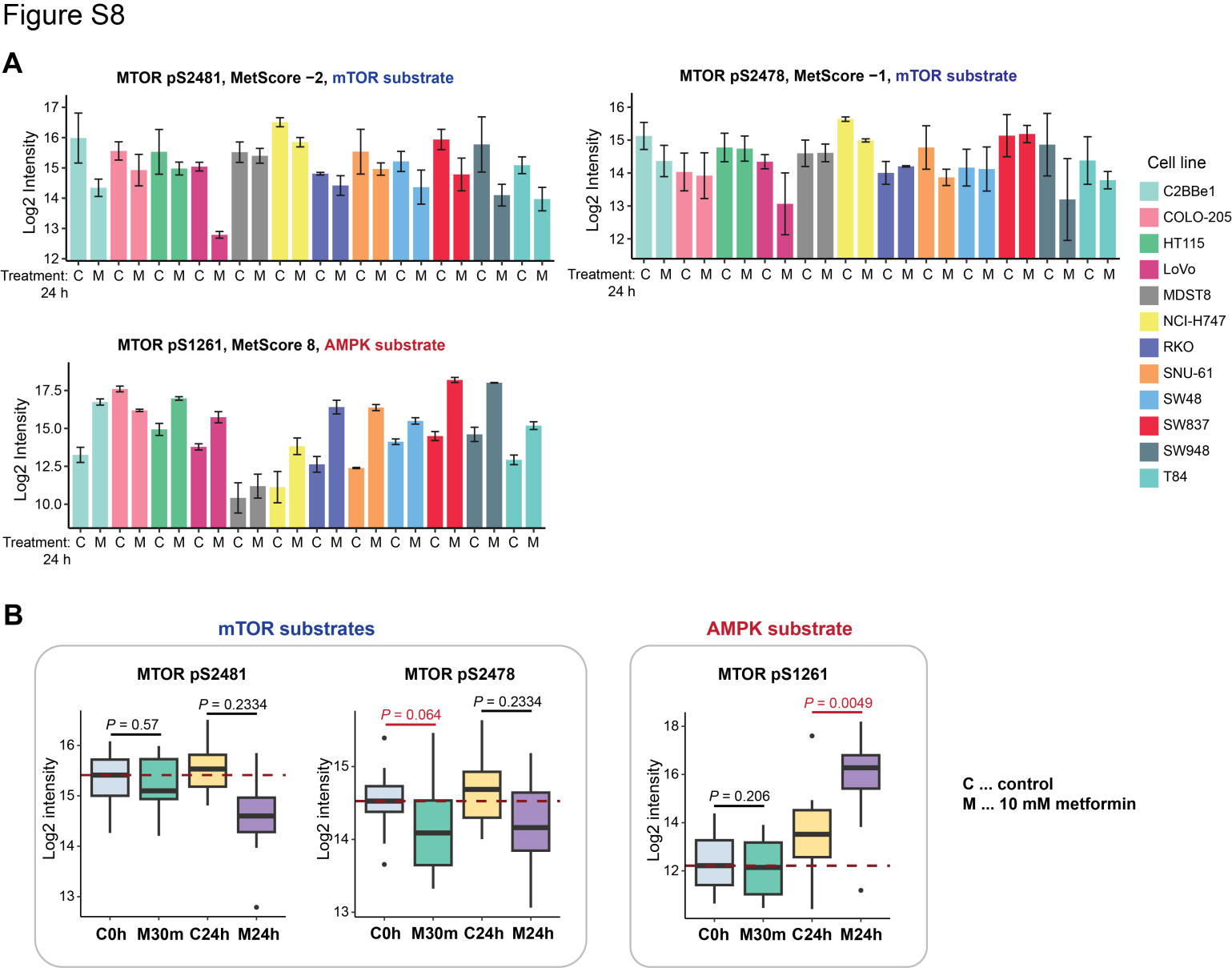


**Figure S8**: **Regulation of mTOR P-sites by metformin (related to** **Figure 2**). **(A)** Phosphorylation of mTOR P-sites that are known mTOR autophosphorylation sites (upper panel) and AMPK targets (lower panel) after 24 hours in untreated control samples (C) or metformin-treated samples (M). **(B)** The boxplots summarize the intensities of the P-sites in panel (A) across the 12 cell lines (n = 12; n = 11 for Ser 1261 C0h) in control 0 h (C0h), control 24 h (C24h), metformin 30 min (M30m), and metformin 24 h (M24h) samples. Statistical analysis was performed using paired Wilcox test.


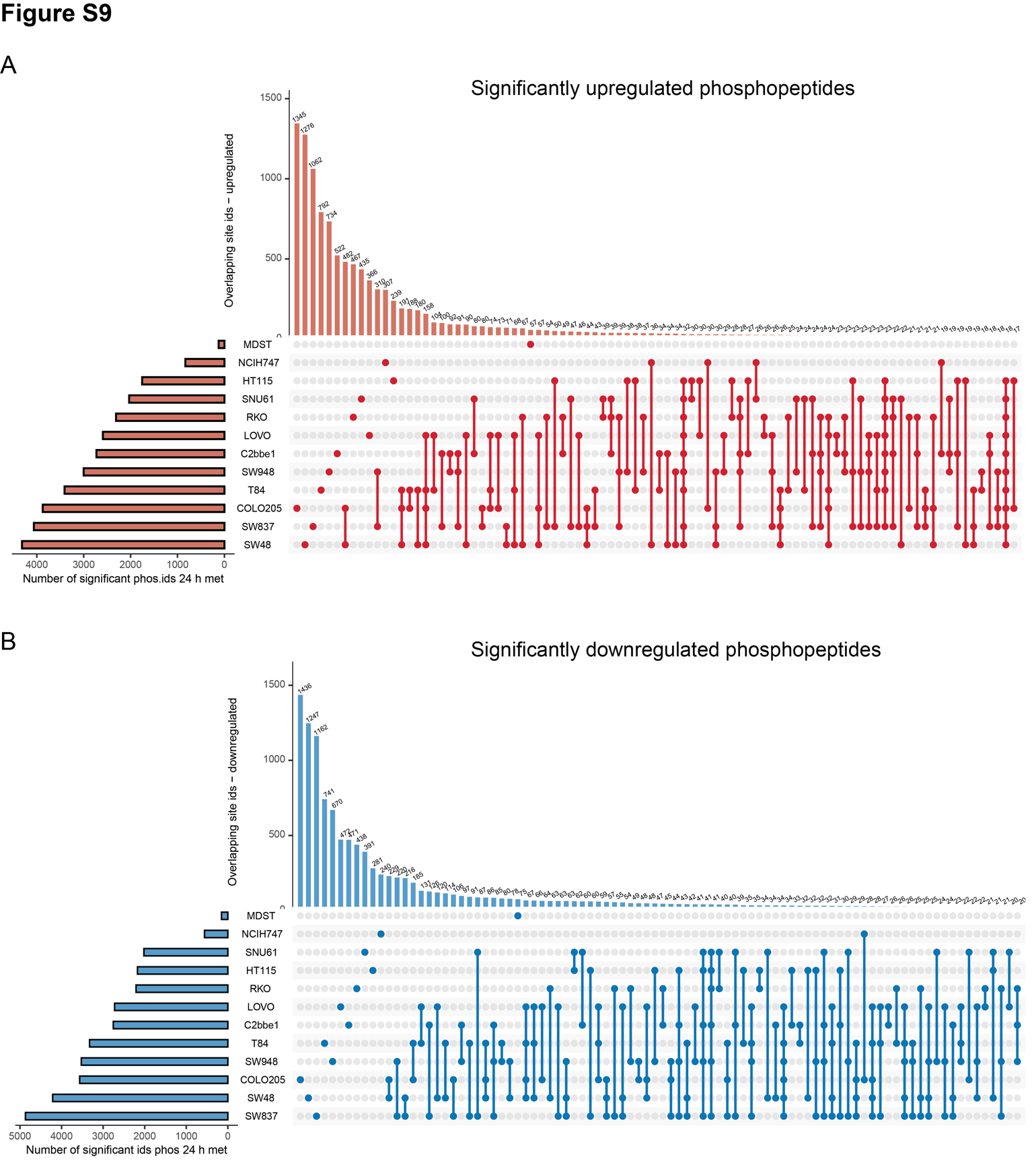


**Figure S9: Cell line-specificity of the phosphoproteome level response at the individual sites level (related to Figure 2). (A-B)** UpSet plots show the overlaps of the significantly up- **(A)** or down- **(B)** regulated phosphopeptides between individual cell lines.

**
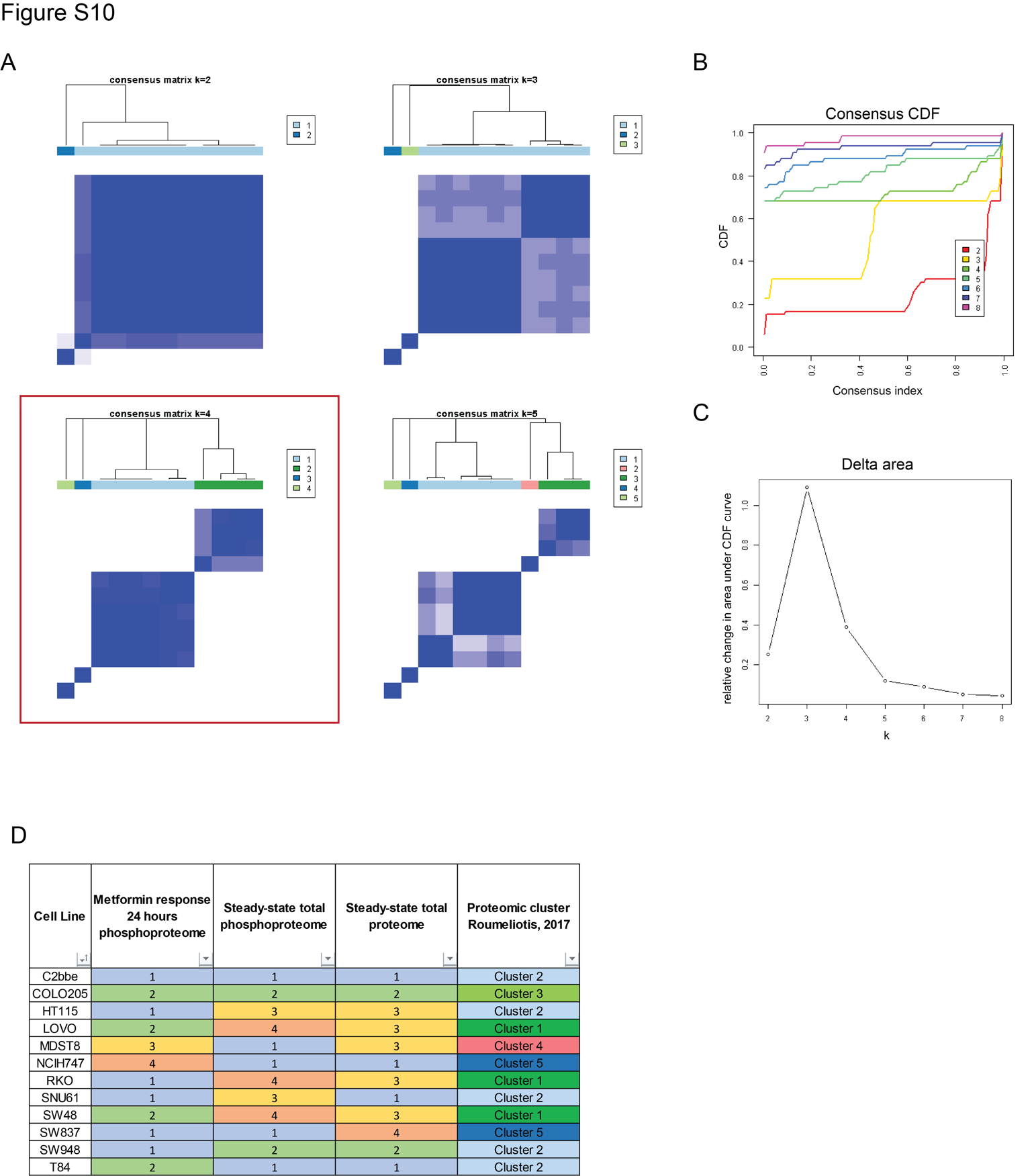
**

**Figure S10: Consensus clustering analysis of the metformin response (related to Figure 2). (A)** Four clusters revealed by the ConsusClusterPlus algorithm based on the top 30% of most variable metformin response (log2 fold change metformin/control after 24 hours) across the 12 cell lines (highlighted by red rectangle). The consensus matrices for target values of k = 2, 3, 4, and 5 are visualized. **(B)** Consensus cumulative distribution function (CDF) plot and **(C)** delta area plot used to determine the number of consensus clusters. **(D)** Clustering based on metformin response mostly does not resemble clusters retrieved based on the steady-state (control) proteome and phosphoproteome.


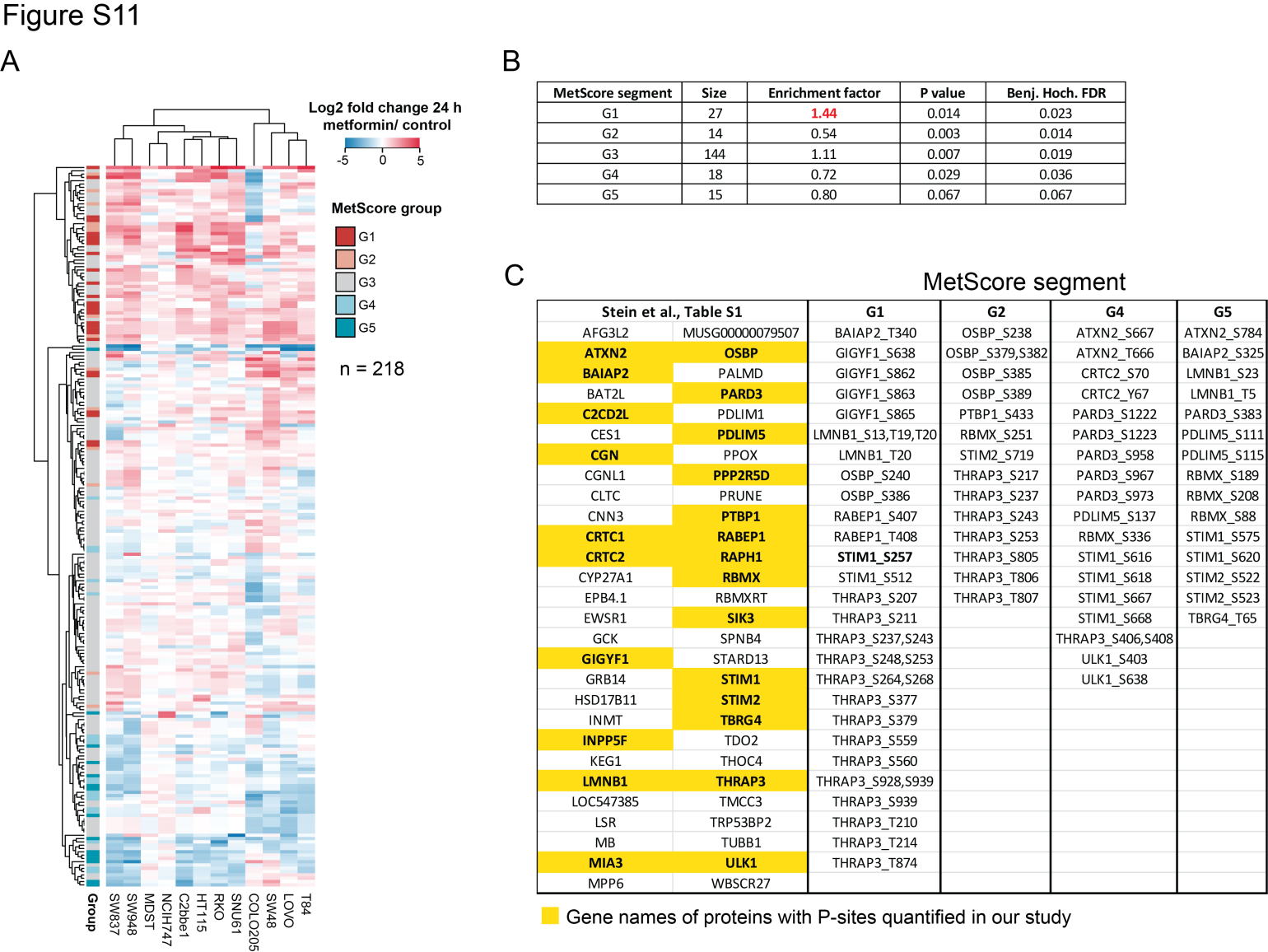


**Figure S11**: **Overlap of the current dataset with the in vivo mouse liver dataset published by Stein et al, (related to** **Figure 4**). **(A)** The 56 phosphoproteins which phosphorylation was increased by metformin in mouse liver and was LKB1/AMPK dependent were matched to the current CRC phosphoproteome dataset (n = 218 phosphopeptides from 24 proteins). The MetScores are indicated. **(B)** Significant overrepresentation (Fisher’s exact test) of the overlapping phosphopeptides in G1 MetScore group. **(C)** The list of 56 phosphoproteins provided by Stein et al is shown. The ids overlapping with our study are highlighted in yellow (n = 24). The phosphopeptides corresponding to the 24 overlapping proteins are listed in their corresponding MetScore G1-G5 segment.

**Figure S12 is provided in a separate supplementary .pdf file**

**Figure S12: PHONEMeS reconstructed signaling networks in individual cell lines (related to Figure 6). (A-L)** The border color corresponds to the protein activity scores calculated using PHONEMeS; the fill color mapping illustrated the decoupleR kinase activity score. The shape indicates whether the node is a P-site measured in our data (ellipse), kinase differentially perturbed in our data (diamond), or protein inferred by the algorithm to be a part of the signaling network (rectangle). The size corresponds to the number of out-going edges.

**
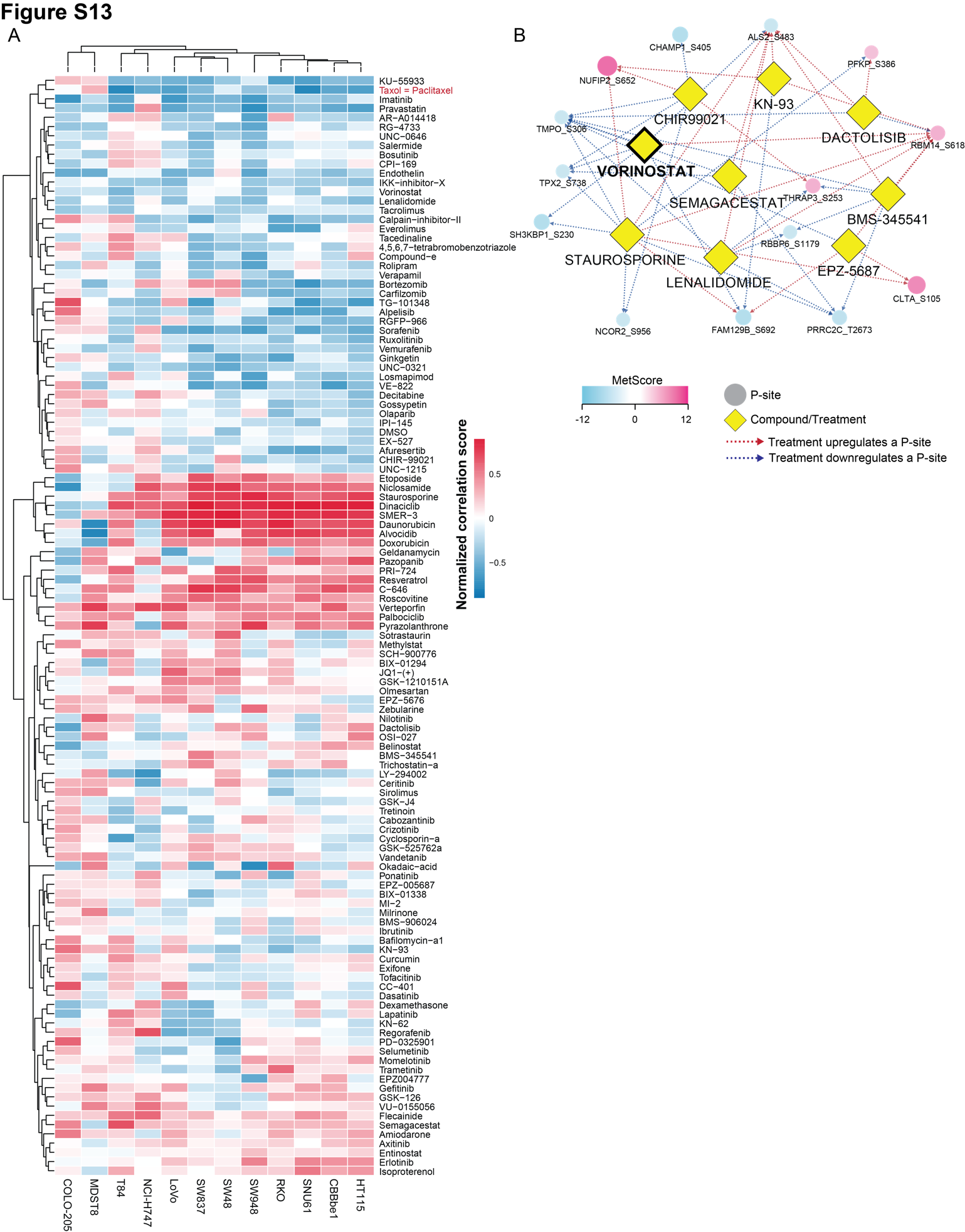
**

**Figure S13: Metformin~drug profile correlation analysis (related to Figure 7). (A)** Heatmap of all metformin~drug profile correlations (normalized correlation score) based on the P100 reduced representative phosphosignature dataset. **(B)** Drugs associated the most with the non-G3 MetScore P-sites based on the PTMsigDB.


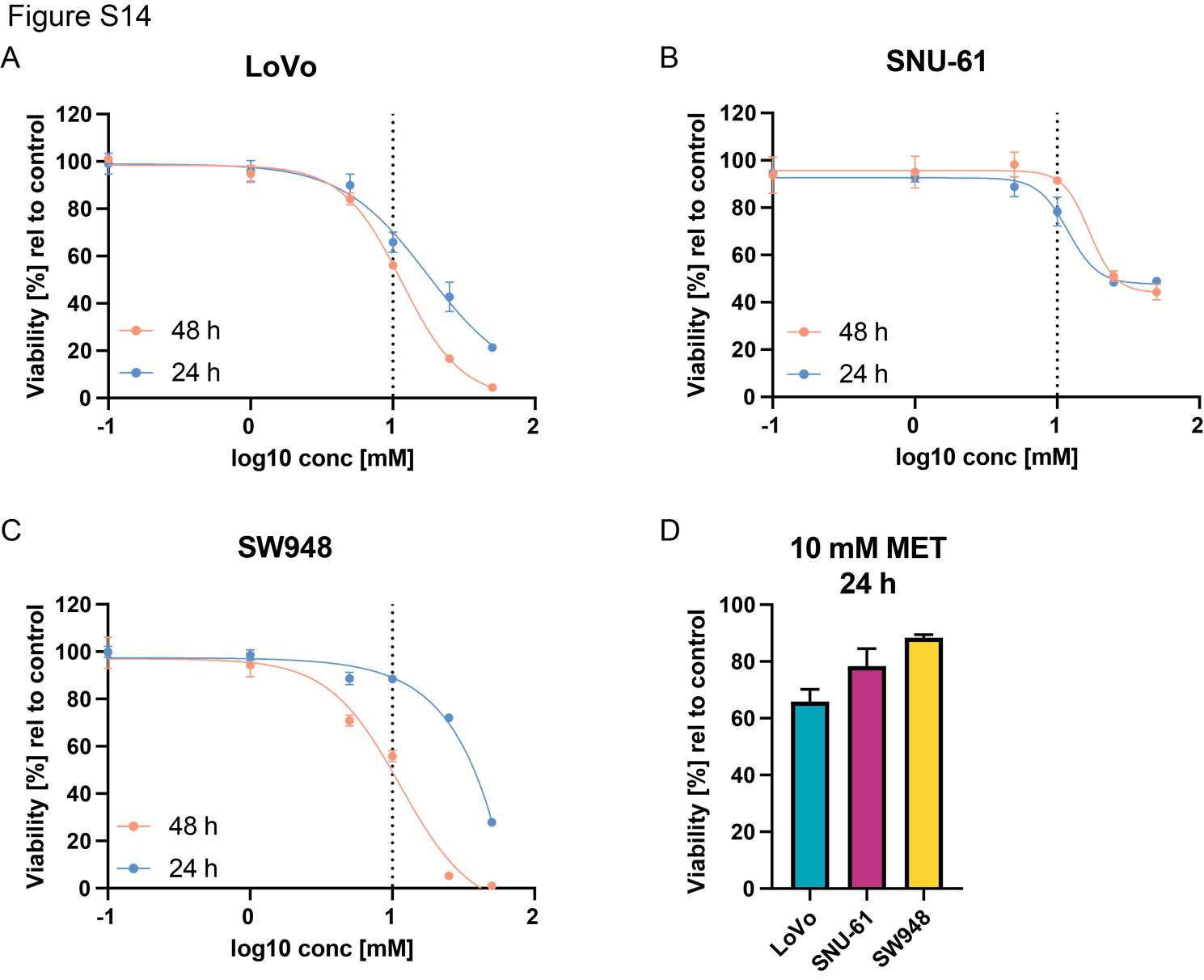


**Figure S14**: **The effect of metformin on cellular proliferation (related to** **Figure 7**). **(A-C)** The effect of metformin in 3 cell lines on cellular proliferation in different concentrations (0.1 mM, 1 mM, 5 mM, 10 mM, 25 mM, and 50 mM) after 24 h and 48 h of cultivation. The curves were fitted in GraphPad Prism version 8.0. **(D)** The effect of 10 mM metformin on proliferation of the three cell lines after 24 hours.

**Table S1: Mutational landscape of the 12 cell CRC cell lines used in this study.** The table was adapted from Iorio et al [^47^](#_ENREF_47), supplementary tables S1E, S2C, S2G, and S2J. MSI – microsatellite instability, CNA – copy number alterations, ns – nonsense, ms – missense, fs – frameshift, if – inframe.

| **Cell line** | **MSI status, (S)table, (L)ow, (H)igh** | **CNA status, (A) - amplification, (D) - deletion** | **genomic variants** | | | | | |
| --- | --- | --- | --- | --- | --- | --- | --- | --- |
|  |  |  | **TP53** | **KRAS** | **BRAF** | **APC** | **SMAD4** | **CTNNB1** |
| **C2BBe1** | **MSS/MSI-L** | STK4 (A) | ns (c.610G>T) |  |  | ns (c.4099C>T) | ms (c.1051G>C) | ms (c.734G>C) |
| **COLO-205** | **MSS/MSI-L** | HSP90AB1,TRERF1 (A); FLT3,WASF3 (A); ING1,IRS2,TFDP1 (A); SMAD4 (D) | if (c.308_33326>TA) |  | ms (c.1799T>A) | fs (c.4666_4667insA) |  |  |
| **HT-115** | **MSS/MSI-L** |  | ns (c.637C>T) |  |  | ms (c.992C>T); ns (c.3587C>A); fs (c.4660_4661insA) |  |  |
| **LoVo** | **MSI-H** |  |  | ms (c.38G>A) |  | ns (c.3340C>T); fs (c.4289delC); ms (c.8447G>A) |  |  |
| **MDST8** | **MSS/MSI-L** | STK4 (A); MET (A) |  |  | ms (c.1799T>A); ms (c.1798G>A) | fs (c.4660_4661insA) |  |  |
| **NCI-H747** | **MSS/MSI-L** | SMAD4 (D) | ms (c.473G>T) | ms (c.38G>A) |  | ns (c.481C>T); ns (c.4285C>T) |  |  |
| **RKO** | **MSI-H** |  |  |  | ms (c.1799T>A) |  |  |  |
| **SNU-61** | **MSS/MSI-L** | MYC (A); SMAD4 (D); APC (D) | ms (c.524G>A) | ms (c.35G>A) |  | ns (c.4348C>T) |  |  |
| **SW48** | **MSI-H** |  |  |  |  | ms (c.8140C>T) |  | ms (c.98C>A) |
| **SW837** | **MSS/MSI-L** | MAP2K4 (D); SMARCA4 (D); ARGAP3 (D); SMAD4 (D) | ms (c.742C>T) | ms (c.34G>T) |  | ns (c.637C>T); ns (c.4348C>T) |  |  |
| **SW948** | **MSS/MSI-L** | FLT3,WASF3 (A); ING1,IRS2,TFDP1 (A) | fs (c.351delG) | ms (c.182A>T) |  | ns (c.3340C>T); ns (c.4285C>T) | ms (c.1609G>T) |  |
| **T84** | **MSS/MSI-L** | CDKN2A (D); SMAD4 (D) | ess_splice (c.560-1G>T) | ms (c.38G>A) |  | fs (c.4464delA) | ms (c.1020G>C) |  |

**Supplementary tables S2-S6 are provided separately as .xlsx files**

**Table S2: Overview of the phosphoproteome relative quantification results.** All phosphoproteome quantification results are provided together with the statistical analysis results as described in **Methods**.

**Table S3: Overview of the total proteome relative quantification results.** All total quantification results are provided together with the statistical analysis results as described in **Methods**.

**Table S4: Proteins and P-sites corresponding to the protein ids included in the “Ma list” (related to Figure 3)**. Data for the “**In-list**” proteins are shown. “**In-list**” – the protein id was identified and confirmed by Ma et al. to be a lysosomal interactor of metformin. Log2 fold changes (metformin/control at 24 hours) across all cell lines are provided.

**Table S5: Stratification of the phosphoproteome based on metformin responsivity: MetScore.** The phosphoproteome quantification results are provided together with the corresponding MetScore and MetScore grouping (see **Figure 4A**). The MetScore was calculated and the phosphoproteome was filtered as described in the **Methods** section.

**Table S6: Kinase activity inference results.** Normalized enrichment scores (NES) estimated using decoupleR R package are provided. Only kinases with at least five measured targets were included in the kinase activity estimation (NA – not enough targets measured). The 3-color scale corresponds to the NES values with blue and red highlighting the negative and positive values of the NES, respectively; white color corresponds to 0.

**Table S7: Overview of literature reporting drug-metformin combination treatments for drugs shown in Figure 7A and B.** MET – metformin.

| Drug Combination | **Author** | **Journal** | **Year** | **Experimental Design** | **MET in vitro concentration used** | **Experimental model** | **Insights and Conclusion** | **PMID** |
| --- | --- | --- | --- | --- | --- | --- | --- | --- |
| MET + doxorubicin | **Iliopoulos et al** | Cancer Research | 2011 | In vitro and in vivo | In vitro: 0.1 mM | Mammary epithelial cells; fibrocystic breast tissue; prostate adenocarcinoma; lung epithelial carcinoma; mouse xenografts | Metformin had comparable effects on tumor regression and preventing relapse when combined with a 4-fold reduced dose of doxorubicin that is not effective as a monotherapy. The combination of metformin and doxorubicin prevented relapse in xenografts generated with prostate and lung cancer cell lines. | 21415163 [^115^](#_ENREF_115) |
| MET + doxorubicin | **Li Y. et al** | Mol Pharm | 2019 | In vitro and in vivo | N.A. | Human breast cancer cell lines (MCF7/ADR - doxorubicin-resistant cell line); mouse xenografts | The dual-drug-loaded liposomes increased tumor targeting and intratumoral blood oxygen saturation, which suggested that the tumor reoxygenation effect of MET facilitated the exertion of its synergistic activity with DOX against MCF7/ADR xenografts | 31095914 [^116^](#_ENREF_116) |
| MET + doxorubicin | **Marinello et al** | Sci Rep | 2019 | In vitro | 6 μM | Human breast cancer cell lines (MCF-7; MDA-MB-231) | Metformin treatment increased sensitivity to doxorubin-induced oxidative stress and apoptosis. | 30971831 [^117^](#_ENREF_117) |
| MET + doxorubicin | **Coronel-Hernandez et al** | Frontiers in Oncology | 2021 | In vitro | 0.001 – 65 mM | Colorectal cancer cell lines | The triple therapy of metformin, doxorubicine, and sodium oxamate reduced cell viability throuh mTOR/AKT pathway inhibition, recovered autophagy suppression in CRC, and promoted apoptosis. | 34123772 [^118^](#_ENREF_118) |
| MET + doxorubicin | **Rocca et al** | Ther Adv Med Oncol | 2021 | Phase II clinical trial | N.A. | HER2-positive, early, or locally advanced breast cancer patients | The concomitant administration of trastuzumab, liposomal doxorubicin, docetaxel, and metformin is safe and shows good activity, but does not appear to improve activity over conventional sequential regimens. | 33613693 [^119^](#_ENREF_119) |
| MET + Imatinib | **Lee et al** | Development and Reproduction | 2017 | In vitro | 0.25–4 mM | Colorectal cancer cell lines | Metformin and imatinib synergistically decreased CRC viability and arrested the cell cycle in S and G2/M phase. | 28785735 [^120^](#_ENREF_120) |
| MET + Imatinib | **Nan et al** | Cancer Lett | 2021 | In vitro and in vivo | In vitro: 5 mM (IC25) | Ewing sarcoma cell lines (TC71, TC32, and A673) and mouse xenografts | Metformin/imatinib drug combination regimen widely suppressed multiple dominant mechanisms in EwS genesis, growth, and metastasis, including key EWS-FLI1 downstream targets that converge into the PI3K/AKT/mTOR signaling pathway. | 31672491 [^121^](#_ENREF_121) |
| MET + Navitoclax | **Levesley et al** | PloS One | 2013 | In vitro | 8 mM | Pediatric Glioma Cells | Navitoclax, along with metformin and 2-deoxyglucose, induced apoptosis more efficiently compared to single reagents. | 23691145 [^90^](#_ENREF_90) |
| MET + Navitoclax | **Li et al** | Molecular Cancer Therapeutics | 2017 | In vitro and in vivo | In vitro: 1-100 mM (10 mM synergetic effect) | Hepatocellular carcinoma, prostate cancer, colorectal cancer, lung cancer, cervical cancer; mouse xenografts | Metformin and navitoclax showed synergy both in vitro and in vivo on p53 defective cells and not on wt p53 cells. | 28533436 [^91^](#_ENREF_91) |
| MET + Nilotinib | **Na et al** | Korean J In | 2021 | In vitro | 0.1 – 10 mM (5 mM drug combination assays) | Chronic myeloid leukemia cells | Treatment with metformin and nilotinib induced 40 to 50% cell death compared to metformin alone. Further, metformin was able to re-sensitize nilotinib-resistant cells by inducing caspase 3 or caspase 9 cleavage. | 32241082 [^122^](#_ENREF_122) |
| MET + Nutlin-3 | **Shimazu et al** | BMC Cancer | 2017 | In vitro | 0-80 mM (10 and 40 mM for drug combination assays) | Malignant mesothelioma | The combination of metformin and nutlin-3a showed synergistic growth inhibitory effects. In cell cycle analyses, the combination also increased sub-G1 fractions better than the reagents alone. | 28464864 [^123^](#_ENREF_123) |
| MET + Paclitaxel | **Hanna et al** | Gynecol Oncol | 2012 | In vitro | 0.5 mM | Endometrial cancer cell lines (ECC-1; Ishikawa) | Metformin potentiated the effects of paclitaxel in endometrial cancer cells through inhibition of cell proliferation and modulation of the mTOR pathway. | 22252099 [^124^](#_ENREF_124) |
| MET + Paclitaxel | **Tseng et all** | Biochem Pharmacol | 2013 | In vitro | 0.1 mM | Nonsquamous non-small cell lung cancer cell lines (H1650 and H1703) | Metformin was able to not only decrease the paclitaxel-induced p38 MAPK-mediated ERCC1 expression, but also augment the cytotoxic effect induced by paclitaxel. | 23228696 [^125^](#_ENREF_125) |
| MET + Paclitaxel | **Lengyel et al** | Am J Obstet Gynecol | 2015 | In vitro and in vivo | In vitro: 10-40 mM | Human ovarian cancer cell lines (SKOV3ip1; HeyA8) cell; mouse xenografts | Metformin altered the metabolism in ovarian cancer cells, prevented tumor growth, and increased sensitivity to chemotherapy in vitro and in mouse models. | 25446664 [^126^](#_ENREF_126) |
| MET + Paclitaxel | **Xiao et al** | AAPS PharmSciTech | 2018 | In vitro and in vivo | N.A. | Human breast cancer cells (4T1); mouse xenografts | In vitro anti-tumor studies demonstrated that metformin and paclitaxel had a synergistic effect and co-delivery micelles induced higher cytotoxicity and apoptosis against 4T1 breast cancer cells than free drugs. | 29869309 [^127^](#_ENREF_127) |
| MET + Paclitaxel | **Yu et al** | Cancer Biotherapy and Radiopharmaceuticals | 2020 | In vitro and in vivo | In vitro: 5-160 μM | Human ovarian cancer cells; mouse xenografts | Metformin was able to increase sensitivity to paclitaxel by regulating autophagy mediated by small nucleolar RNA host gene 7 in ovarian cancer cells. | 32522016 [^128^](#_ENREF_128) |
| MET + Pazopanib | **Fiala et al** | Cancer Manag Res | 2021 | Clinical study | N.A. | Clinical data from patients with metastatic renal cell carcinoma | The use of pazopanib or sunitinib along with metformin seemed to increase the progression-free survival and overall survival of metastatic renal cell carcinoma patients. | 34054309 [^129^](#_ENREF_129) |
| MET + SN-38 | **Hu et al** | Sci Rep | 2014 | In vitro and in vivo | In vitro: 0.1 mM | Human breast epithelial ductal carcinoma; serous ovarian adenocarcinoma | Low-dose metformin or SN-38 may reprogram cancer cells into non-cancerous cells in a FOXO3-dependent manner. Treatment of metformin or SN-38 was found to decrease ovarian tumor growth in mice. | 25056111 [^130^](#_ENREF_130) |
| MET + Vorinostat | **Chen et al** | Oncotarget | 2017 | In vitro | 1 mM | Human lung adenocarcinoma | Vorinostat combined with gefitinib increased sensitivity to EGFR-TKI resistant cells. When metformin was added, it aided in the sensitivity increase because it inhibits anti-apoptotic proteins. | 29212192 [^131^](#_ENREF_131) |
| MET + Niclosamide | **Kang et al** | Cancers (Basel) | 2021 | In vitro and in vivo | In vitro: 10 mM | Human colorectal cancer cell lines (SW480, DLD-1); patient-derived organoids from colon cancer; mouse xenografts | Using patient-derived cancer organoid and an APC-MIN mice model, it was found that the metformin and niclosamide combination was effective for APC-mutated CRC. | 34298652 [^132^](#_ENREF_132) |
| MET + Resveratrol | **Cheng et al** | Free Radic Biol Med | 2022 | In vitro | 5-40 mM (20 mM for drug combination assays) | Triple-negative human breast cancer cell lines (MDA-MB-231) | Metformin inhibited the proliferation and migration induced by low dose resveratrol and enhanced the anti-Triple-negative breast cancer activity of high dose resveratrol. | 35038549 [^133^](#_ENREF_133) |
| MET + Sorafenib | **Chen et al** | Oncol Rep | 2015 | In vitro | 5 μM | Anaplastic thyroid carcinoma cells ( HTh74 and doxorubicin resistant HTh74Rdox) | Sorafenib and metformin synergistically decreased the proliferation of anaplastic thyroid carcinoma cell lines and the outgrowth of their derived cancer stem cells. A combined treatment enabled a significant dose reduction of sorafenib. | 25683253 [^134^](#_ENREF_134) |
| MET + Sorafenib | **Chung et al** | Ann Hepatobiliary Pancreat Surg | 2018 | In vitro | 5-10 mM | Hepatocellular carcinoma cell lines (HepG2.2.15, patient-derived graft HCC cell lines) | The study demonstrated cytotoxic effects of metformin and synergistic antitumor effects of sorafenib in hepatocellular carcinoma cells in vitro. | 30215039 [^135^](#_ENREF_135) |
| MET + Sorafenib | **Guo et al** | Cancer Sci | 2016 | In vitro and in vivo | In vitro: 10 mM | Hepatocellular carcinoma cell lines (MHCC97H); mice xenografts | The data showed that the combination of sorafenib and metformin inhibited proliferation and invasion in vitro, prolonged median survival, and reduced lung metastasis of HCC in vivo. | 26752068 [^136^](#_ENREF_136) |
| MET + Sorafenib | **Wang et al** | Leuk Res | 2015 | In vitro | 0.2-16 mM (4 mM for drug combination assays) | Acute myeloid leukemia cell lines (MV4-11 and THP-1) | In the presence of metformin, the anticancer potential of sorafenib was found to be synergistically enhanced with the remarkably reduced protein expression of the mTOR/p70S6K/4EBP1 pathway. | 26505133 [^137^](#_ENREF_137) |
| MET + Sorafenib | **You et al** | J Hematol Oncol | 2016 | In vitro and in vivo | In vitro: 5-50 mM (10 mM for the drug combination assays) | Human hepatocellular carcinoma cell line (MHCC97H) and mouse xenografts | Metformin in combination with sorafenib suppressed cell proliferation, promoted cell apoptosis, and suppressed epithelial-mesenchymal transition process both in vitro and in vivo. Metformin in combination with sorafenib significantly minimized postoperative recurrence and lung metastasis of HCC in orthotopic mouse model. | 26957312 [^138^](#_ENREF_138) |
| MET + Etoposide | **Teixeira et al** | J Bras Pneumol | 2013 | In vitro | 15-30 mM | Non-small cell lung cancer cell line ( NCI-H460) | The use of metformin as monotherapy reduced the metabolic viability of the cell line studied. Combining metformin with cisplatin or etoposide produced a synergistic effect and was more effective than was the use of cisplatin or etoposide as monotherapy. | 24473757 [^139^](#_ENREF_139) |

**References**

114. Perez-Riverol Y, Csordas A, Bai J, et al. The PRIDE database and related tools and resources in 2019: improving support for quantification data. *Nucleic Acids Res*. Jan 8 2019;47(D1):D442-D450. doi:10.1093/nar/gky1106

115. Iliopoulos D, Hirsch HA, Struhl K. Metformin decreases the dose of chemotherapy for prolonging tumor remission in mouse xenografts involving multiple cancer cell types. *Cancer Res*. May 1 2011;71(9):3196-201. doi:10.1158/0008-5472.CAN-10-3471

116. Li Y, Luo J, Lin MT, et al. Co-Delivery of Metformin Enhances the Antimultidrug Resistant Tumor Effect of Doxorubicin by Improving Hypoxic Tumor Microenvironment. *Mol Pharm*. Jul 1 2019;16(7):2966-2979. doi:10.1021/acs.molpharmaceut.9b00199

117. Marinello PC, Panis C, Silva TNX, et al. Metformin prevention of doxorubicin resistance in MCF-7 and MDA-MB-231 involves oxidative stress generation and modulation of cell adaptation genes. *Scientific reports*. Apr 10 2019;9(1):5864. doi:10.1038/s41598-019-42357-w

118. Coronel-Hernandez J, Salgado-Garcia R, Cantu-De Leon D, et al. Combination of Metformin, Sodium Oxamate and Doxorubicin Induces Apoptosis and Autophagy in Colorectal Cancer Cells via Downregulation HIF-1alpha. *Frontiers in oncology*. 2021;11:594200. doi:10.3389/fonc.2021.594200

119. Rocca A, Cortesi P, Cortesi L, et al. Phase II study of liposomal doxorubicin, docetaxel and trastuzumab in combination with metformin as neoadjuvant therapy for HER2-positive breast cancer. *Ther Adv Med Oncol*. 2021;13:1758835920985632. doi:10.1177/1758835920985632

120. Lee J, Park D, Lee Y. Metformin Synergistically Potentiates the Antitumor Effects of Imatinib in Colorectal Cancer Cells. *Dev Reprod*. Jun 2017;21(2):139-150. doi:10.12717/DR.2017.21.2.139

121. Nan X, Wang J, Cheng H, et al. Imatinib revives the therapeutic potential of metformin on ewing sarcoma by attenuating tumor hypoxic response and inhibiting convergent signaling pathways. *Cancer Lett*. Jan 28 2020;469:195-206. doi:10.1016/j.canlet.2019.10.034

122. Na YJ, Yu ES, Kim DS, Lee DH, Oh SC, Choi CW. Metformin enhances the cytotoxic effect of nilotinib and overcomes nilotinib resistance in chronic myeloid leukemia cells. *Korean J Intern Med*. Mar 2021;36(Suppl 1):S196-S206. doi:10.3904/kjim.2019.336

123. Shimazu K, Tada Y, Morinaga T, et al. Metformin produces growth inhibitory effects in combination with nutlin-3a on malignant mesothelioma through a cross-talk between mTOR and p53 pathways. *BMC Cancer*. May 2 2017;17(1):309. doi:10.1186/s12885-017-3300-y

124. Hanna RK, Zhou C, Malloy KM, et al. Metformin potentiates the effects of paclitaxel in endometrial cancer cells through inhibition of cell proliferation and modulation of the mTOR pathway. *Gynecol Oncol*. May 2012;125(2):458-69. doi:10.1016/j.ygyno.2012.01.009

125. Tseng SC, Huang YC, Chen HJ, et al. Metformin-mediated downregulation of p38 mitogen-activated protein kinase-dependent excision repair cross-complementing 1 decreases DNA repair capacity and sensitizes human lung cancer cells to paclitaxel. *Biochem Pharmacol*. Feb 15 2013;85(4):583-94. doi:10.1016/j.bcp.2012.12.001

126. Lengyel E, Litchfield LM, Mitra AK, et al. Metformin inhibits ovarian cancer growth and increases sensitivity to paclitaxel in mouse models. *Am J Obstet Gynecol*. Apr 2015;212(4):479 e1-479 e10. doi:10.1016/j.ajog.2014.10.026

127. Xiao Y, Wang S, Zong Q, Yin Z. Co-delivery of Metformin and Paclitaxel Via Folate-Modified pH-Sensitive Micelles for Enhanced Anti-tumor Efficacy. *AAPS PharmSciTech*. Jul 2018;19(5):2395-2406. doi:10.1208/s12249-018-1070-8

128. Yu Z, Wang Y, Wang B, Zhai J. Metformin Affects Paclitaxel Sensitivity of Ovarian Cancer Cells Through Autophagy Mediated by Long Noncoding RNASNHG7/miR-3127-5p Axis. *Cancer Biother Radiopharm*. Jun 9 2020;doi:10.1089/cbr.2019.3390

129. Fiala O, Ostasov P, Rozsypalova A, et al. Metformin Use and the Outcome of Metastatic Renal Cell Carcinoma Treated with Sunitinib or Pazopanib. *Cancer management and research*. 2021;13:4077-4086. doi:10.2147/CMAR.S305321

130. Hu T, Chung YM, Guan M, et al. Reprogramming ovarian and breast cancer cells into non-cancerous cells by low-dose metformin or SN-38 through FOXO3 activation. *Scientific reports*. Jul 24 2014;4:5810. doi:10.1038/srep05810

131. Chen H, Wang Y, Lin C, et al. Vorinostat and metformin sensitize EGFR-TKI resistant NSCLC cells via BIM-dependent apoptosis induction. *Oncotarget*. Nov 7 2017;8(55):93825-93838. doi:10.18632/oncotarget.21225

132. Kang HE, Seo Y, Yun JS, et al. Metformin and Niclosamide Synergistically Suppress Wnt and YAP in APC-Mutated Colorectal Cancer. *Cancers*. Jul 9 2021;13(14)doi:10.3390/cancers13143437

133. Cheng T, Wang C, Lu Q, et al. Metformin inhibits the tumor-promoting effect of low-dose resveratrol, and enhances the anti-tumor activity of high-dose resveratrol by increasing its reducibility in triple negative breast cancer. *Free Radic Biol Med*. Feb 20 2022;180:108-120. doi:10.1016/j.freeradbiomed.2022.01.010

134. Chen G, Nicula D, Renko K, Derwahl M. Synergistic anti-proliferative effect of metformin and sorafenib on growth of anaplastic thyroid cancer cells and their stem cells. *Oncol Rep*. Apr 2015;33(4):1994-2000. doi:10.3892/or.2015.3805

135. Chung YG, Tak E, Hwang S, et al. Synergistic effect of metformin on sorafenib in in vitro study using hepatocellular carcinoma cell lines. *Ann Hepatobiliary Pancreat Surg*. Aug 2018;22(3):179-184. doi:10.14701/ahbps.2018.22.3.179

136. Guo Z, Cao M, You A, et al. Metformin inhibits the prometastatic effect of sorafenib in hepatocellular carcinoma by upregulating the expression of TIP30. *Cancer Sci*. Apr 2016;107(4):507-13. doi:10.1111/cas.12885

137. Wang F, Liu Z, Zeng J, et al. Metformin synergistically sensitizes FLT3-ITD-positive acute myeloid leukemia to sorafenib by promoting mTOR-mediated apoptosis and autophagy. *Leuk Res*. Dec 2015;39(12):1421-7. doi:10.1016/j.leukres.2015.09.016

138. You A, Cao M, Guo Z, et al. Metformin sensitizes sorafenib to inhibit postoperative recurrence and metastasis of hepatocellular carcinoma in orthotopic mouse models. *J Hematol Oncol*. Mar 8 2016;9:20. doi:10.1186/s13045-016-0253-6

139. Teixeira SF, Guimaraes Idos S, Madeira KP, Daltoe RD, Silva IV, Rangel LB. Metformin synergistically enhances antiproliferative effects of cisplatin and etoposide in NCI-H460 human lung cancer cells. *J Bras Pneumol*. Nov-Dec 2013;39(6):644-9. doi:10.1590/S1806-37132013000600002

### Data availability

The mass spectrometry data have been all deposited to the ProteomeXchange Consortium via the PRIDE [^114^](#_ENREF_114) PXD036826. (To review the dataset please go to https://www.ebi.ac.uk/pride/login, and use the following login details: **Username:** reviewer_pxd036826@ebi.ac.uk ; **Password:**Cv5mW5oh ). The entire data basis can be also interactively explored at (<https://yslproteomics.shinyapps.io/Metformin/>). The website also contains the full versions of the supplementary tables **Table S2** and **Table S3**.
