# Supplementary material for: Phosphoproteomic analysis of metformin signaling in colorectal cancer cells elucidates mechanism of action and potential therapeutic opportunities
Source: Clin Transl Med. 2023 Feb 13;13(2):e1179. doi: 10.1002/ctm2.1179 (PMC9925373; doi:10.1002/ctm2.1179)

Figure S12A

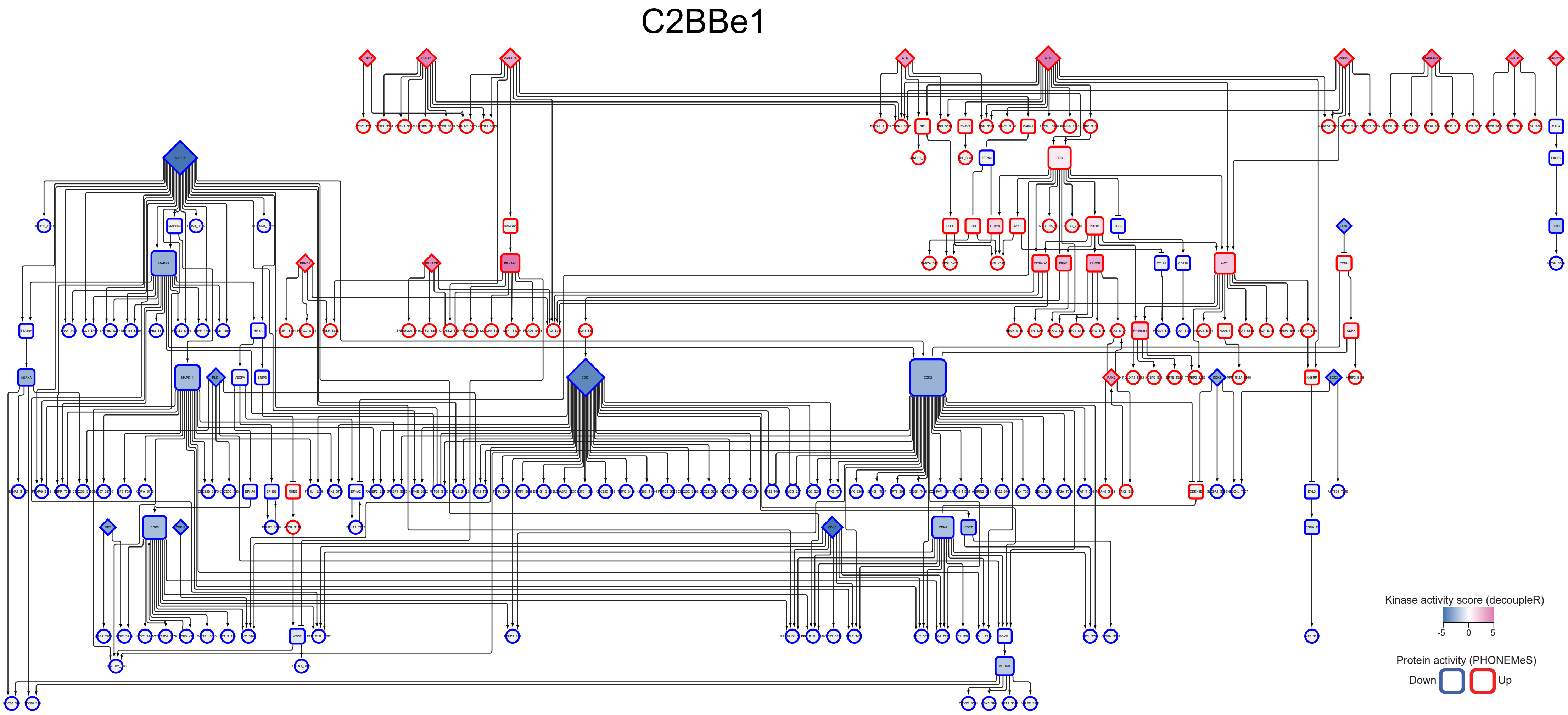

Figure S12: PHONEMeS reconstructed signaling networks in individual cell lines (related to Figure 6). (A-L) The border color corresponds to the protein activity scores calculated using PHONEMeS; the fill color mapping illustrated the decoupleR kinase activity score. The shape indicates whether the node is a P-site measured in our data (ellipse), kinase differentially perturbed in our data (diamond), or protein inferred by the algorithm to be a part of the signaling network (rectangle). The size corresponds to the number of out-going edges.

Figure S12B

COLO 205

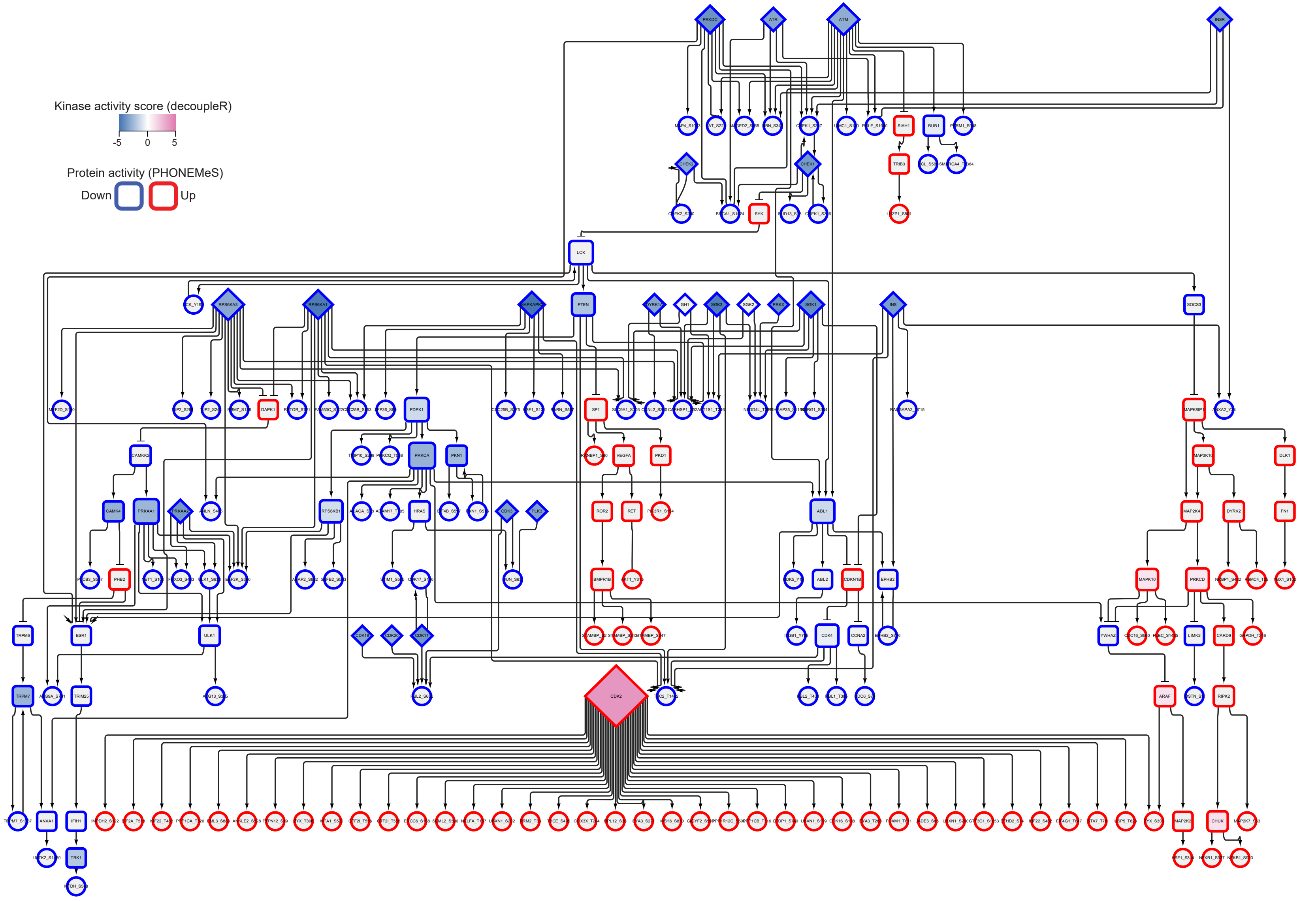

Figure S12C

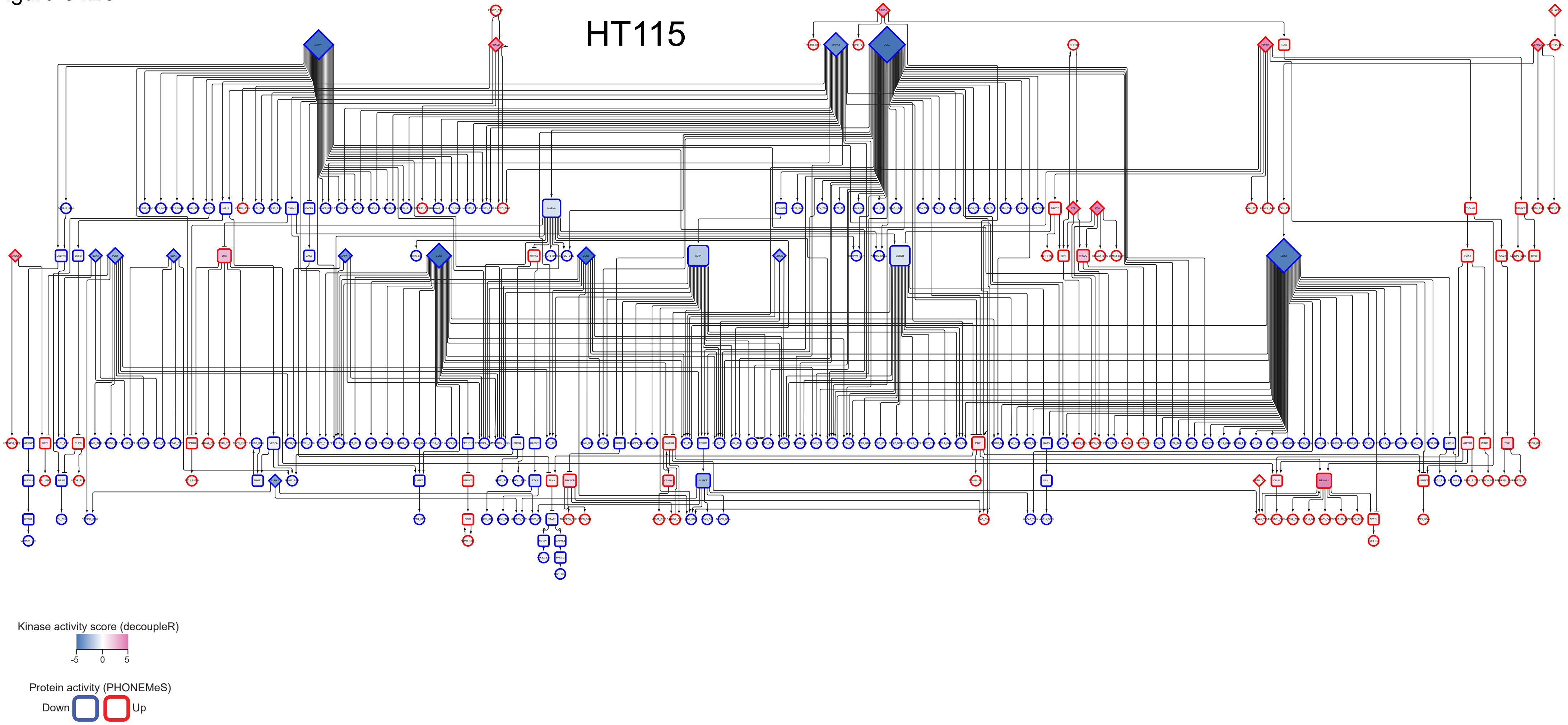
HT115
Kinase activity score (decoupleR)
-5
0
5
Protein activity (PHONEMeS)
Down
Up

Figure S12D

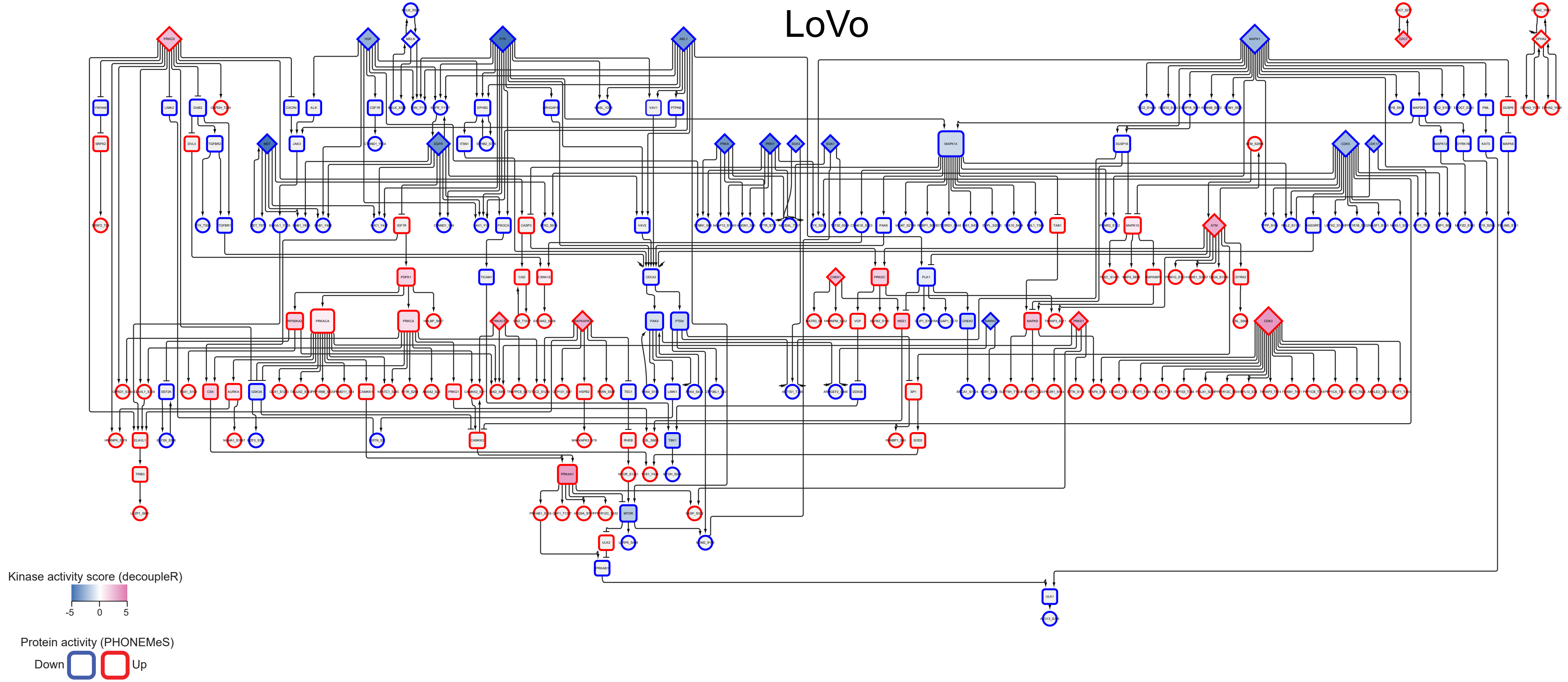
LoVo
Kinase activity score (decoupleR)
-5
0
5
Protein activity (PHONEMeS)
Down
Up

Figure S12E

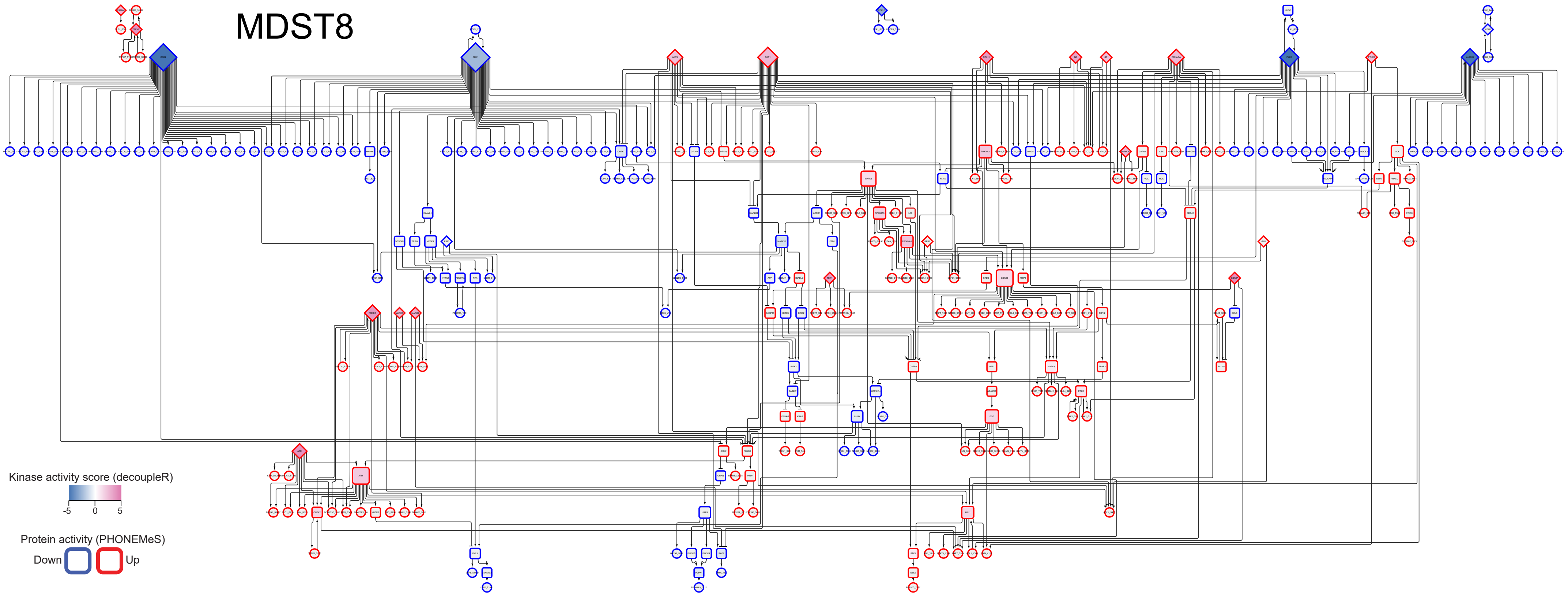
MDST8
Kinase activity score (decoupleR)
-5
0
5
Protein activity (PHONEMeS)
Down
Up

Figure S12F

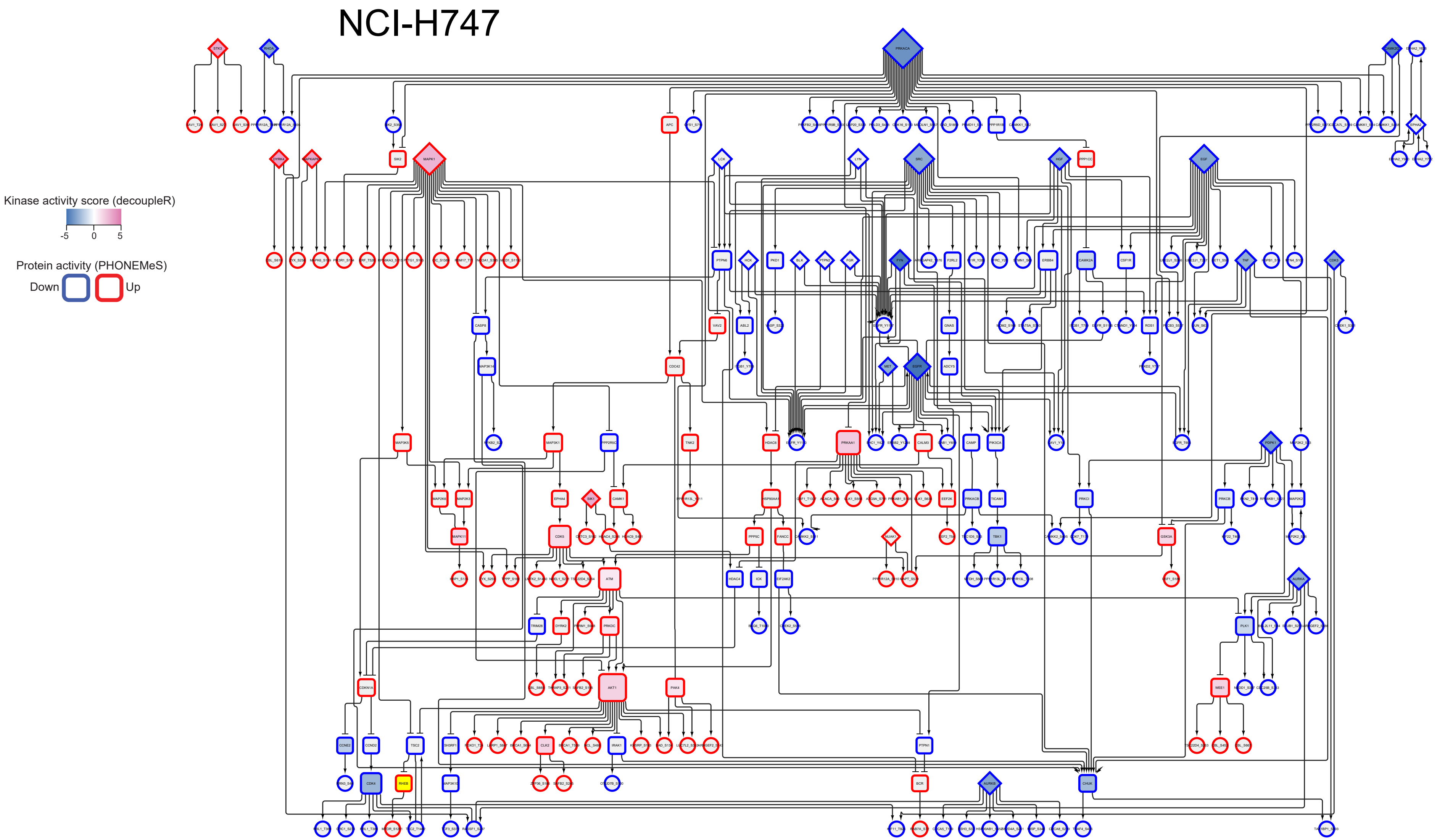
NCI-H747
Kinase activity score (decoupleR)
-5
0
5
Protein activity (PHONEMeS)
Down
Up

Figure S12G

RKO

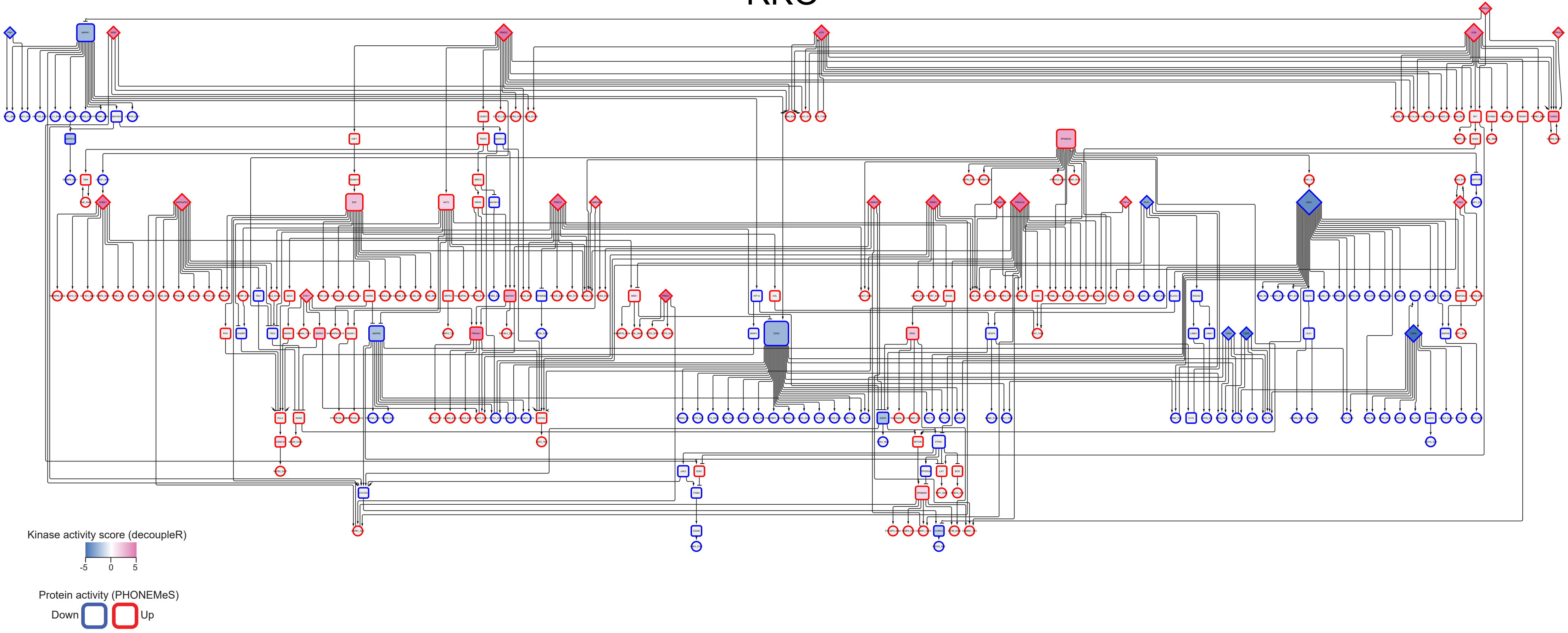

Supplementary Figure 6H

SNU-61

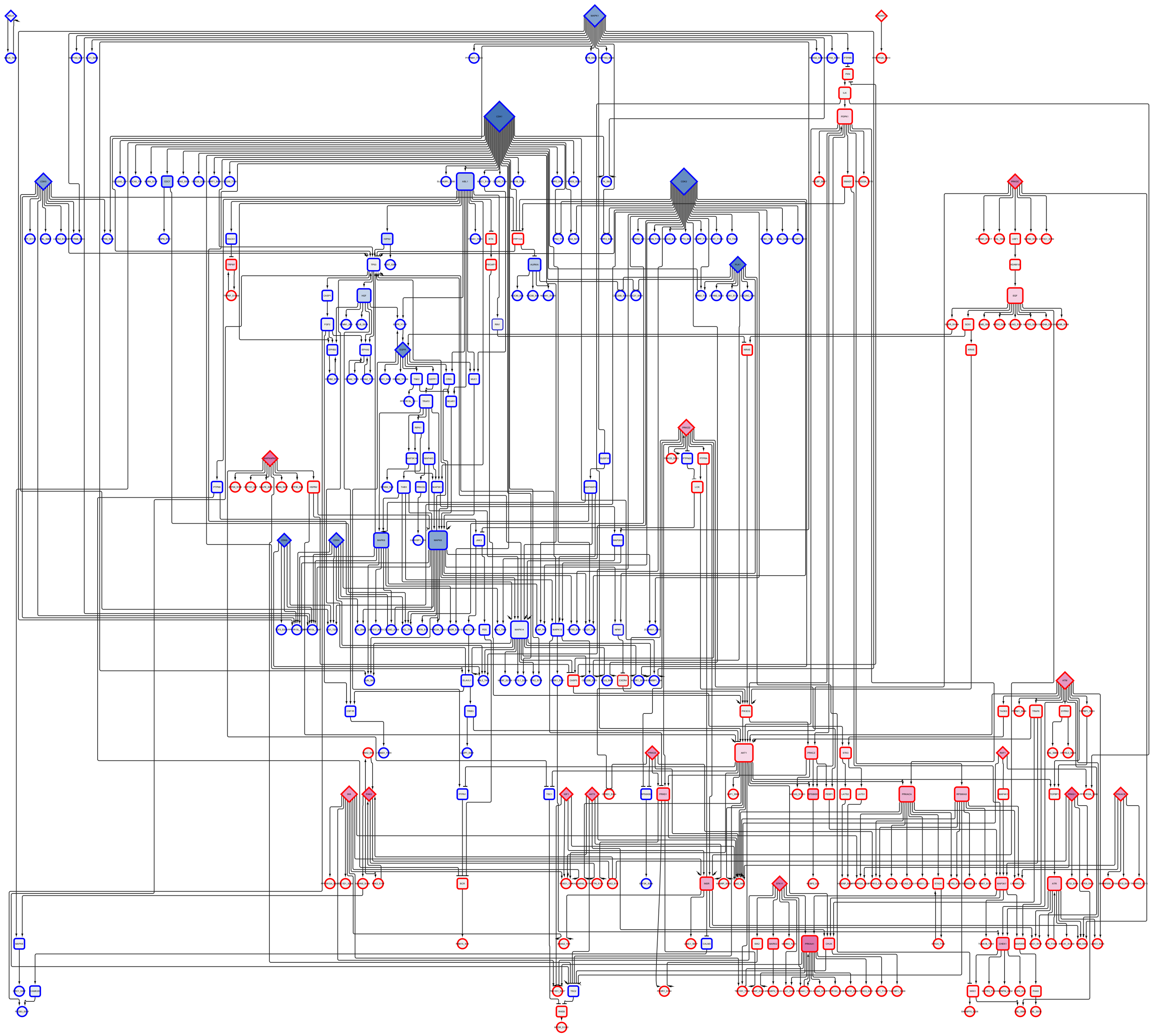

Figure S12I

SW48

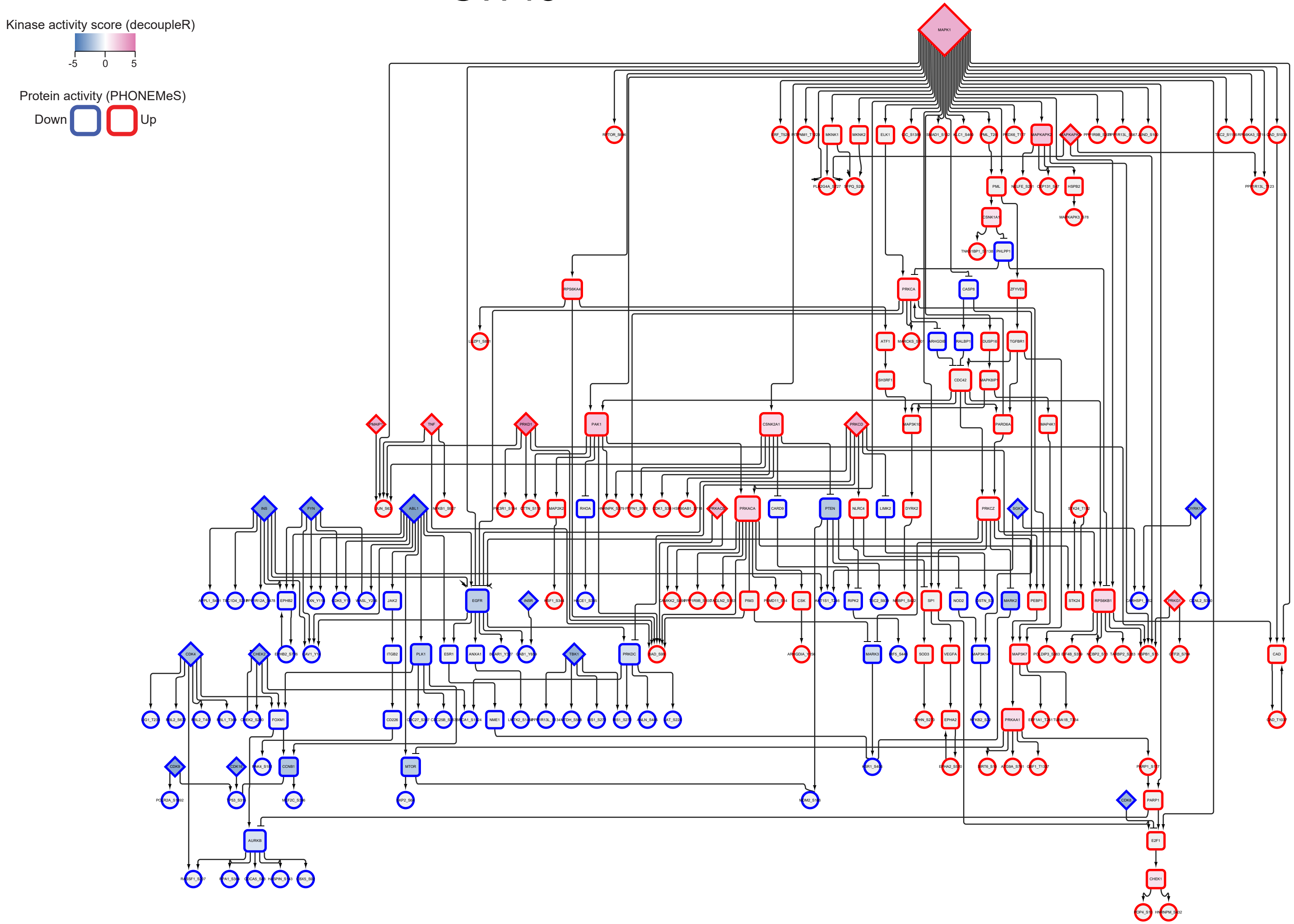

Figure S12J

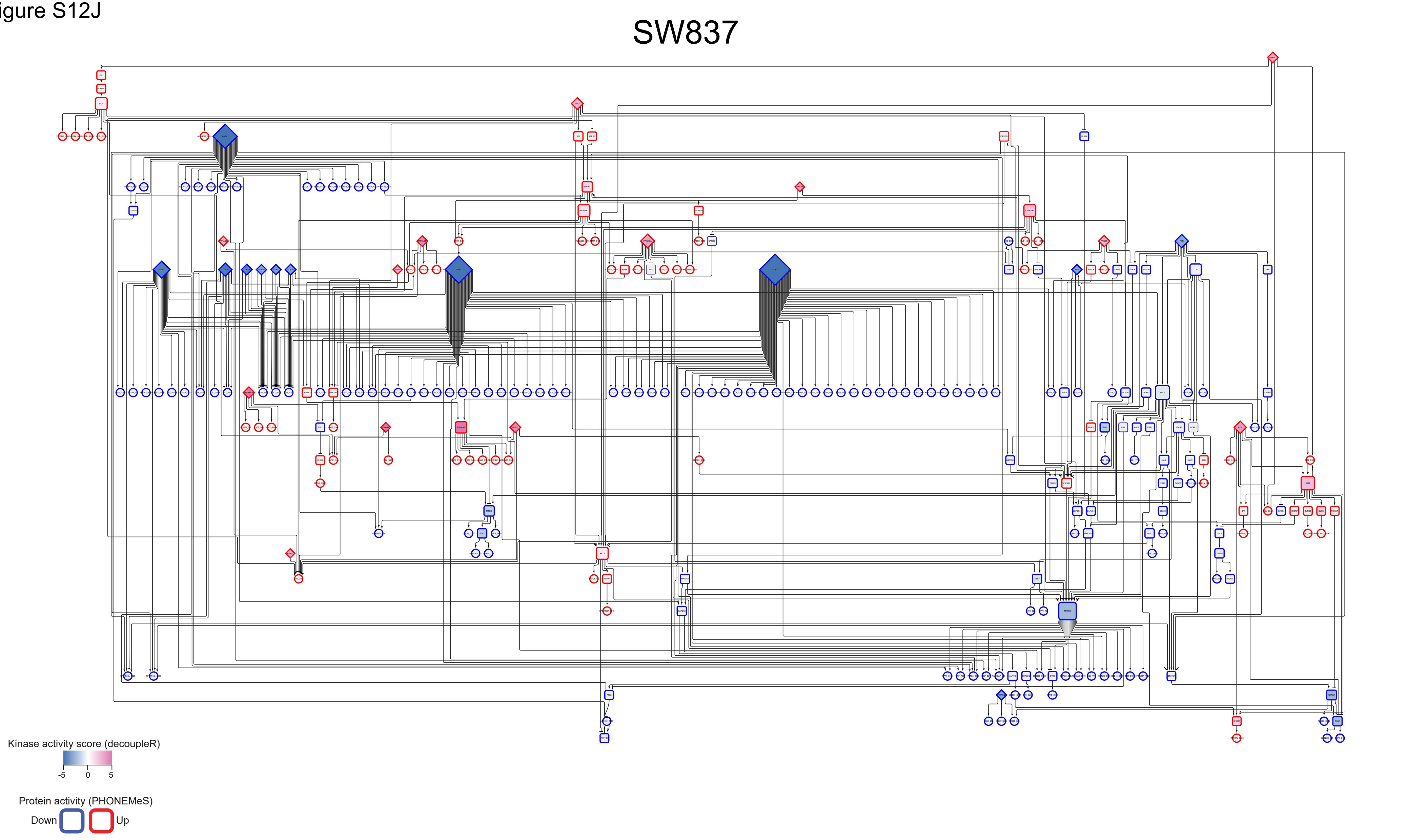
SW837
Kinase activity score (decoupleR)
-5
0
5
Protein activity (PHONEMeS)
Down
Up

Figure S12K

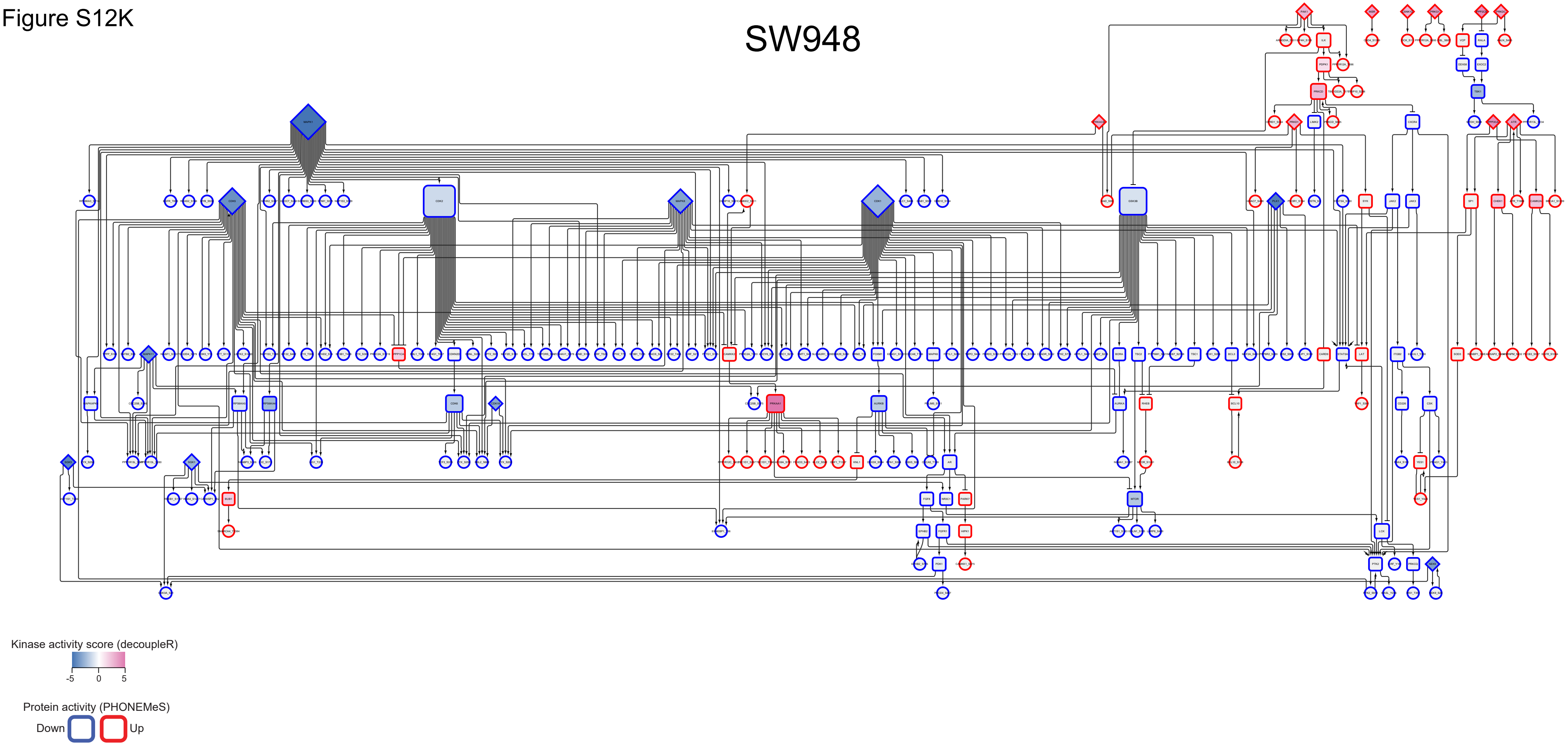
SW948
Kinase activity score (decoupleR)
-5
0
5
Protein activity (PHONEMeS)
Down
Up

Figure S12L

T84

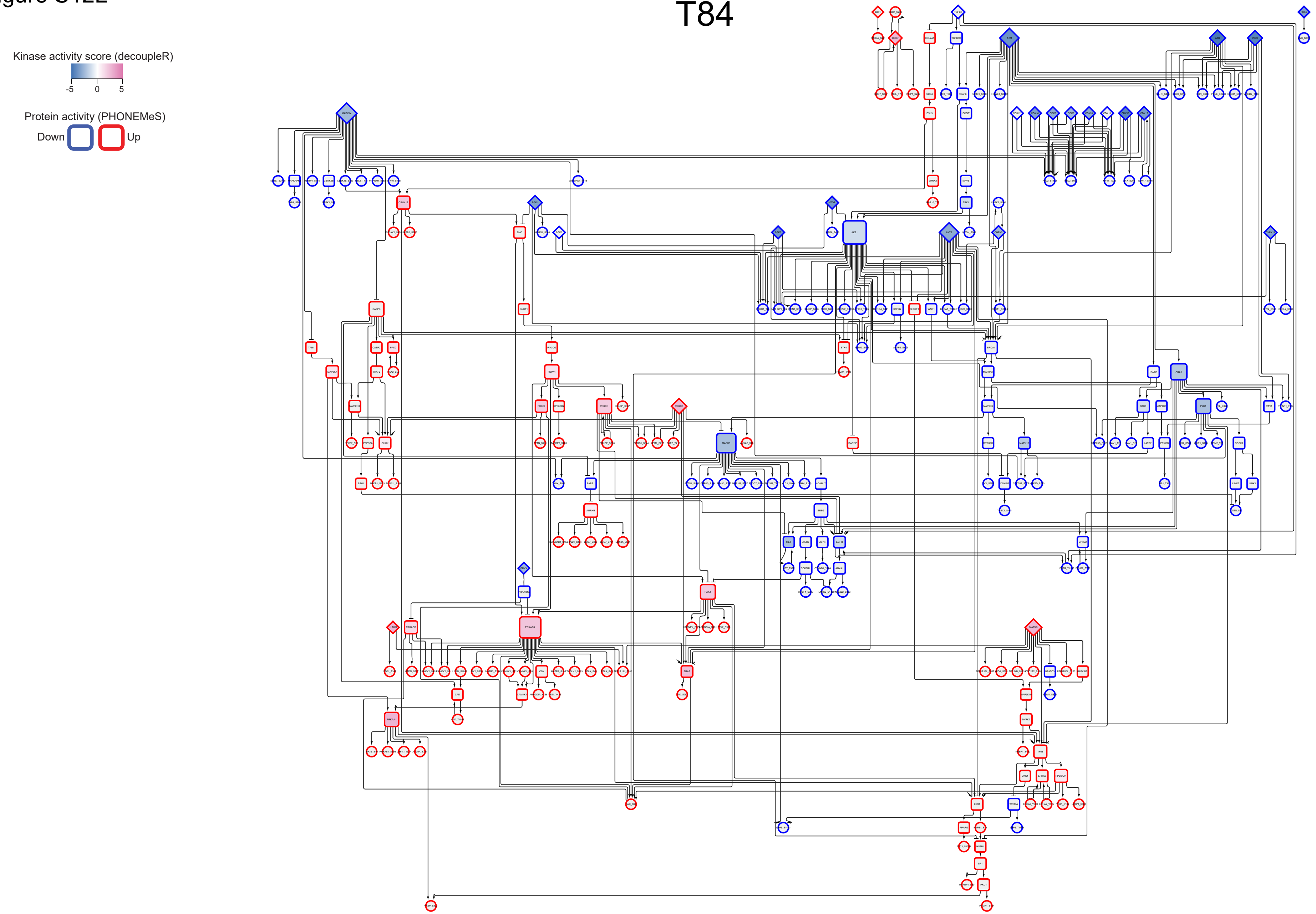

Supplement: Supplementary file 7 — Supporting Information [file CTM2-13-e1179-s002.pdf]
